# Non-model model organisms

**DOI:** 10.1186/s12915-017-0391-5

**Published:** 2017-06-29

**Authors:** James J. Russell, Julie A. Theriot, Pranidhi Sood, Wallace F. Marshall, Laura F. Landweber, Lillian Fritz-Laylin, Jessica K. Polka, Snezhana Oliferenko, Therese Gerbich, Amy Gladfelter, James Umen, Magdalena Bezanilla, Madeline A. Lancaster, Shuonan He, Matthew C. Gibson, Bob Goldstein, Elly M. Tanaka, Chi-Kuo Hu, Anne Brunet

**Affiliations:** 1 Department of Biology, Howard Hughes Medical Institute Stanford University, Stanford, CA 94305 USA; 2 Departments of Biochemistry and of Microbiology & Immunology, Howard Hughes Medical Institute Stanford University, Stanford, CA 94305 USA; 3 Department of Biochemistry & Biophysics, University of California San Francisco, 600 16th St, San Francisco, CA 94158 USA; 4 Departments of Biochemistry & Molecular Biophysics and Biological Sciences, Columbia University, New York, NY 10032 USA; 5 Department of Biology, University of Massachusetts, Amherst, MA 01003 USA; 6 Visiting Scholar, Whitehead Institute, 9 Cambridge Center, Cambridge, MA 02142 USA; 7 The Francis Crick Institute, 1 Midland Road, London, NW1 1AT UK; 8 Randall Division of Cell and Molecular Biophysics, New Hunt’s House, Guy’s Campus, King’s College London, London, SE1 1UL UK; 9 516 Fordham Hall, University of North Carolina Chapel Hill, Chapel Hill, NC 27514 USA; 10 Donald Danforth Plant Science Center, 975 N. Warson Rd, St. Louis, MO 63132 USA; 11 MRC Laboratory of Molecular Biology, Cambridge Biomedical Campus, Francis Crick Avenue, CB2 0QH Cambridge, UK; 12 Stowers Institute for Medical Research, Kansas City, MO 64110 USA; 13 Department of Anatomy and Cell Biology, The University of Kansas School of Medicine, Kansas City, KS 66160 USA; 14 Biology Department, The University of North Carolina at Chapel Hill, Chapel Hill, NC 27599 USA; 15 Research Institute of Molecular Pathology (IMP), Vienna Biocenter (VBC), Campus Vienna Biocenter 1, 1030 Vienna, Austria; 16 Department of Genetics, Stanford University, Stanford, CA 94305 USA; 17 Glenn Laboratories for the Biology of Aging at Stanford, Stanford, CA 94305 USA

## Abstract

Model organisms are widely used in research as accessible and convenient systems to study a particular area or question in biology. Traditionally only a handful of organisms have been widely studied, but modern research tools are enabling researchers to extend the set of model organisms to include less-studied and more unusual systems. This Forum highlights a range of 'non-model model organisms' as emerging systems for tackling questions across the whole spectrum of biology (and beyond), the opportunities and challenges, and the outlook for the future.

## Introduction—model organisms for understanding biology

### Wallace F. Marshall

The transition in biology from description to mechanistic understanding during the 20th century was due in large part to a conscious decision to employ model organisms. The idea of a model organism is that if one wants to study a particular aspect of biology, it makes sense to employ a simple, tractable organism that facilitates experimental work. Bacteriophage, bacteria, corn, and yeast revealed most of what we know about basic molecular biology of the central dogma, while flies, worms, *Arabidopsis*, and mice played a similar role in the study of development. The choice of these systems was not arbitrary—they typically were chosen because they were smaller, simpler, and faster growing than more complex organisms such as humans or trees. The term “model organism” was used to indicate a simplified, tractable system that could be used to study a larger theme of biology, and indicated not so much a feature of the system itself, as an attitude on the part of the researcher. The “phage group” was not primarily interested in how bacteriophage worked as an end in itself, but rather as a means to a larger end of understanding gene regulation. Bacteriophage were simply a convenient model for studying the bigger question. An experiment could be done hundreds or thousands of times more quickly and cheaply using bacteriophage than human cells, so it is hardly surprising that research in simpler systems rapidly outpaced work in humans. Likewise, flies have been studied for a century not so much because so many people find flies themselves interesting, but because flies made genetic analysis of development easy and fast. In some cases the simplest systems are so simple that they lack key features of interest—for example, bacteria and bacteriophage do not employ the full range of regulatory mechanisms that eukaryotes do—requiring the use of more complex model systems such as yeast for the study of chromatin, meiosis, and other eukaryotic-specific parts of the central dogma. The term model organism was used to describe these systems and conveyed the meaning of “an organism that is inherently convenient to study a particular area of biology”.

Because these model organisms were so convenient, and made progress so rapid, researchers flocked to use them. This led to the development of tools and resources specifically for these organisms. Resources include infrastructure, such as databases and strain collections, as well as molecular toolkits and extensive collections of techniques and methods, accumulated over the years by legions of researchers. The development of these resources happened for model organisms because so many people were working on them, and because they were already so convenient. Why spend time developing methods for a less convenient system? As a result, model organisms began to outpace other systems not only in terms of their inherent convenience, but also in terms of the availability of infrastructure to study them. This difference was highlighted by the early genome projects, which for obvious reasons focused on model organisms. Once the yeast, *Drosophila* and *Caenorhabditis elegans* genomes were available, it made even less sense to work on anything else. The gap in methodology and resources between the select few model organisms and everything else led to a gradual linguistic shift in how the term “model organism” was understood, so that now many people, when they say model organism, use it not in its original sense, but instead in the sense of “an organism for which a wealth of tools and resources exist”.

But it was always appreciated that the major model organisms, while convenient for studying many aspects of biology, weren’t necessarily the best systems for all possible questions. None of the standard models were that good at regenerating, for example, and the extremely sparse coverage of biodiversity represented by standard models meant that evolutionary questions had to be handled very carefully. Model organisms were known for many of these hard-to-reach areas of biology, but they were only model organisms in the original sense (convenient for the study of a biological process) but not in the newer sense (possessing infrastructure and resources). Fortunately, the continual decrease in cost of genomic sequencing has now made it feasible to determine a genome sequence for these classic but under-supported models. Even if, as is often the case, established genome centers refuse to take on a new organism, citing lack of a large community of researchers, it is now possible for individual labs to assemble their own sequences. Once a genome sequence is in hand, many methods, such as RNA sequencing, can be immediately applied, and other methods such as CRISPR come into range for development. As a result, there has been an explosion of interest in extending the set of model organisms to include both classic systems long known to be excellent models for particular areas of biology, as well as completely novel systems that have never been explored experimentally but which pose fascinating challenges for mechanistic understanding. We will refer to organisms that are models in the original sense, but not yet in the newer sense, as “non-model model organisms” (NMMO).

The present Forum describes the opportunities created by several such non-model model organisms, as well as the challenges faced in developing methods and resources to study them. The use of genomic information is a common thread, as is the emphasis on Biology writ large. The organisms discussed here were picked up because of their inherent advantages for studying key biological questions, including pattern formation (diatoms, *Stentor*), branching morphogenesis (*Physcomitrella*, *Ashbya*), regeneration (*Stentor*, axolotl), and aging (killifish). The diversity of life addressable using NMMO provides new opportunities for studying the evolution of multicellular life (*Volvox*), body plans (*Nematostella*, tardigrades, cerebral organoids), and cell biological processes (*Oxytricha*, *Naegleria*, *Physcomitrella*, fission yeasts). Other questions now being asked using NMMO are more on the sci-fi end of the spectrum, including suspended animation (tardigrades, killfish), phase transitions (*Ashbya*), and nanobiotechnology (R bodies, diatoms). All of these examples have one thing in common—exploiting the unique biological features of a special organism to address questions of general importance. These organisms aren’t being studied because they are weird, or because of a fondness for biodiversity, but because they make it easier to ask central questions about biology that have remained unanswered to this day.

## Diatoms are ready for their close-up

### James J. Russell and Julie A. Theriot

**Fig. 1:**
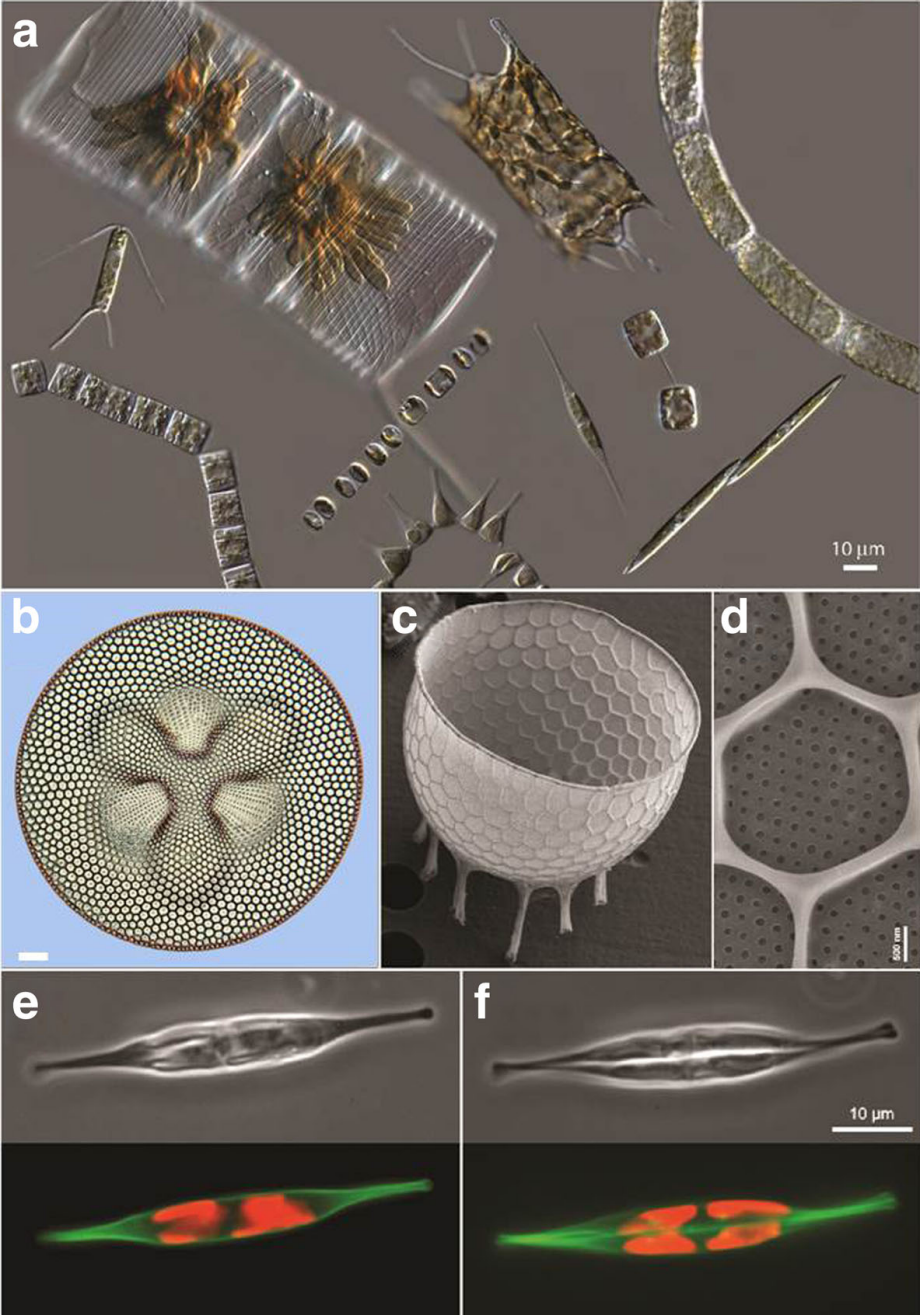
Images of various diatom species. **a** Differential interference contrast images of (clockwise from *top left*): *Striatella unipunctata*, *Odontella* sp., *Stephanopyxis turris*, *Pseudo-nitzschia* sp., *Thalassiosira* sp., *Cylindrotheca* sp., *Asterionellopsis glacialis*, *Skeletonema costatum*, *Grammatophora oceanica*, and *Chaetoceros* sp. Images are courtesy of Colleen Durkin and reproduced from [324]. **b** Differential interference contrast image of *Coscinodiscus excavatus,* image courtesy of Robert Lavigne. **c**–**d** Scanning electron micrographs of *Stephanopyxis turris* theca (**c**) and nanoscale features (**d**), images courtesy of Mark Webber. **e**-**f** *Cylindrotheca fusiformis* before cell division (**e**) and during cell division (**f**). *Top*: phase contrast. *Bottom*: polymerized silica labeled with HCK-123 dye (*green*) and endogenous chlorophyll fluorescence (*red*). *Scale bar* in **b** 20 μm

Diatoms are unicellular eukaryotes abundant in aquatic environments. Their photosynthesis represents a significant fraction of global primary productivity and oceanic carbon sequestration [1]. Among cell biologists, however, diatoms are best known for their extraordinary and beautifully nano-patterned cell walls, made of silicon dioxide—that is, glass [2] (Fig. 1a). Many of us first encountered diatoms in the form of isolated glass cell walls, known as frustules, mounted on slides used as a measure of resolving power, for dark field alignment of microscopes, or in scanning electron micrographs invoking alien-like architecture (Fig. 1b–d). Their exquisite complexity is reminiscent of high-magnification images of snowflakes; however, the diatom frustules are created by genetically encoded developmental programs, and as such are highly reproducible and characteristic for many of the 10,000 to 100,000 estimated species [3]. The variety found even in a single environmental sample can be sufficient to inspire endlessly fascinating but very tiny art [4]. How do these cells design and build their glass houses?

A wide variety of organisms, including protozoa such as radiolarians, many vascular plants, and even some metazoans such as the hexatinellid sponges, have independently developed the ability to precipitate silicon dioxide from soluble silicon compounds (for example silicic acid) in water [5], in a process analogous to the more familiar biomineralization processes used by humans and other vertebrates to precipitate calcium phosphate in our bony skeletons, or by mollusks to make shells using calcium carbonate. In all these cases, the inorganic material is carefully organized and patterned by active cellular processes, and organic molecules are intimately intertwined with the minerals in ways that enhance their material properties and determine their characteristic larger-scale architectures [6, 7]. For diatoms, the fundamental building block of the glass frustule is a near-spherical silicon dioxide nodule about 40 nm in diameter [8]. These precipitated nodules can be formed from soluble silicic acid by several characterized diatom proteins, notably the silaffins [9]. However, the mechanisms by which the diatom cells assemble these simple structural precursors into highly regular nanoscale and microscale patterns in the valve of the frustule are largely unknown. While subcellular microtubule and actin distributions show intriguing correlations with some frustule features [10] and pharmacological disruption of microtubules can lead to defects in pattern determination [11], there is essentially no molecular information available about the mechanisms of pattern formation.

Why do we know so little about the cell biological mechanisms of these lovely organisms? One major barrier has been the lack of useful classic genetics in any diatom species. All characterized diatoms grow vegetatively with diploid genomes, making random mutagenesis strategies difficult, and while individuals of many species have been observed to undergo a sexual cycle in nature [2], they have proved to be shy about reliably mating in the test tube. Despite the lack of classic genetics, several recent advances have made possible the examination of cell biological questions in diatoms using reverse genetic and post-genomic approaches. The first complete genome sequences for two widely cultivated diatoms, *Thalassiosira pseudonana* and *Phaeodactylum tricornutum*, were released in 2004 and 2008, respectively [12, 13], and several additional sequenced diatom genomes have been annotated and made publicly available, belonging primarily to species difficult to culture [14, 15]. Although diatoms are phylogenetically distant from the opisthokonts, including fungi and metazoans, where we have developed our most complete understanding of the mechanisms of regulation of eukaryotic gene expression, nevertheless the structure of diatom genomes appears to be sufficiently similar to our familiar model species that it has been possible to generate robustly annotated genomes, with support from diatom EST libraries and RNA-sequencing data.

Critically, several model diatom species have been shown to be genetically transformable by either electroporation [16] or bacterial conjugation [17], and capable of expressing tagged transgenic protein constructs, including GFP fused to integral components of the glass frustule [18]. In addition, CRISPR-Cas9 genome editing has recently become feasible in model diatoms [19, 20], and several diatom viruses have been sequenced [21] which may provide useful resources for building tools as so many animal viruses have done before them. Owing to the relative ease of adapting these modern genetic tools to diatoms, several labs are engaging additional tools with promising results, including proximity proteomics, live cell microscopy, and super-resolution fluorescence microscopy [22].

What is next for the study of pattern formation in diatoms? Unfortunately, the two model diatoms whose genomes have been robustly annotated are not among the more charismatic of this clade. They are both small and structurally simple; indeed *Phaeodactylum tricornutum* is poorly silicified and does not produce clear nano-patterns, and the tiny valve of *Thalassiosira pseudonana* displays only a rudimentary silica pore structure. A few more annotated genomes for a few elaborately structured but still rapidly growing laboratory strains would be particularly useful; one enticing candidate is *Cylindrotheca fusiformis*, a large (~50 μm length), motile diatom with gracious long arms and a dramatic helical twist along its valvar (cell division) axis (Fig. 1e). *C. fusiformis* is amenable to electroporation-mediated genetic transformation (unpublished data). In addition to the intrinsic value of diatoms as a case for studying pattern formation and biomineralization, diatoms have also attracted attention as sources for large-scale biomolecule production (including lipids for fuel or nutrition) [23], and further development of molecular methods for diatoms could enable genetic optimization for this purpose. Diatoms are easy to grow and wondrous to observe, and now is an ideal time to apply modern approaches to reexamine the ancient mystery of how diatoms achieve their nanoscale elegance.

## *Stentor coeruleus* as a model for single-cell regeneration

### Pranidhi Sood and Wallace F. Marshall

Individual cells can exhibit a great deal of cellular complexity in the organization of subcellular features and organelles. These subcellular patterns must be established and maintained to ensure a cell functions properly—for example, the apico-basal polarity of epithelial cells is required for them to correctly organize in sheets [24]. Cells are not small and amorphous, therefore, but can display complex and invariable internal organization. In fact, some even rival the size and complexity of multicellular embryos. How is morphological complexity created and regulated within a single membrane bound sac of cytoplasm?

**Fig. 2:**
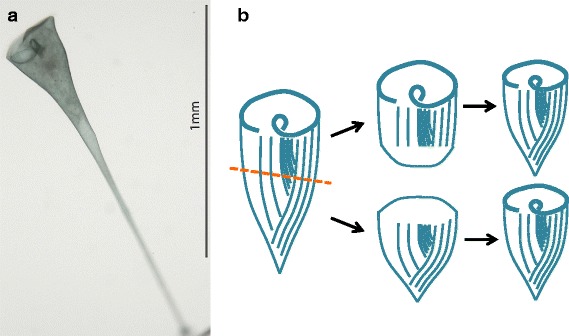
Single-cell regeneration in *Stentor coeruleus* **a** A living Stentor cell. The oral apparatus, located towards the *upper left* of the image, is a large ring of cilia that collects food particles from the surrounding pond water. At the other end of the cell, a holdfast attaches the cell to the surface of pond plants. **b** Regeneration after bisection of a Stentor cell. The panel on the *left* shows the longitudinal strips of blue pigment that serve as markers for cellular pattern. When a Stentor cell is cut in half with a glass needle, as indicated by the *dotted orange line*, each half initially heals its wounds to prevent cytoplasm from leaking out (*middle panels*) and then within approximately one day, regenerates a complete cell (*right panel*), with the anterior half regenerating a new holdfast, and the poster half regenerating a new oral apparatus. Both halves are able to regenerate because the cell contains a long polyploid macronucleus running down the length of the cell, such that when a cell is cut, both halves retain many copies of the genome

Understanding how analogously complex structures arise in multicellular organisms formed the basis of the field of developmental biology. To study these problems before the availability of genetic tools, early researchers took advantage of systems that could regenerate, for example, the planarian flatworm. Similarly, studying the regeneration of cells can provide a window into the origins of cell geometry by decoupling assembly of structures from the normal growth processes in the cell. Historically, the analysis of regeneration in cells was in large part carried out using the giant ciliate *Stentor coeruleus* (Stentor) as an experimental system (Fig. 2). Stentor is a freshwater pond organism, notable for its bright blue coloration and the fact that a single cell can grow to be well over a millimeter in length. Each cell has an invariable, complex anatomy with an oral apparatus at one end, a holdfast at the other, and longitudinal stripes of blue pigment, separated by rows of cilia subtended by microtubule bundles, running down the length of the cell.

Stentor has many advantages for the study of regeneration at the single-cell level. First, it has unrivalled abilities to heal wounds, allowing the cell to recover from massive perturbations. For example, if a cell is bisected, each half will regenerate a normal cell [25]. The ability to sustain and recover from very large wounds is accompanied by the ability to graft pieces back together [26]. Such cellular scale “cut-and-paste” experiments are reminiscent of those that drove the field of experimental embryology. A comprehensive review of the experimental surgical work in Stentor was provided by Tartar [27]. An equally important feature of Stentor is the fact that its prominent organelles provide a clearly visible, built-in coordinate system. For example, a cell’s entire surface is covered with visible features including long, oriented blue pigment stripes. These provide a frame of reference to determine if a cell has been correctly re-formed or if different parts of the cell are in the correct relative positions. Without these naturally occurring fiducial markers, it would be far more difficult to assess the progress of regeneration. It is known that the nucleus [27, 28] and transcription [29–32] are required for most regeneration processes in Stentor. However, there have been comparatively few molecular studies of regeneration in Stentor, owing in part to the challenges of growing large quantities. Stentor divides with a doubling time of several days, and it can take a long time to grow biochemically useful quantities.

Modern genomic technologies remove the need for growing huge numbers of cells and these tools can potentially shed light on the molecular mechanisms of regeneration. The key pre-requisite is to have the genome sequence. This was a major challenge in developing Stentor as a model system, because genome centers and sequencing programs proved unwilling to sequence an organism that didn’t already have a large community of researchers studying it. In the end, we took a DIY approach, sequencing and assembling the genome in our own lab in collaboration with the DeRisi lab at UCSF, and then enlisting a team of experts to analyze the resulting genome. Through teamwork, we were recently able to publish the first Stentor genome [33].

With the Stentor genome in hand, we can begin to decipher the molecular networks behind cellular level regeneration, using techniques such as RNA-seq. We know that transcription is a key requirement for regeneration from foundational biochemical studies [29–32, 34], though specific transcripts driving regeneration were not identified. Also, there is evidence that transcripts synthesized during regeneration can become physically associated with newly formed organelles [32, 35], suggesting that RNA localization might play a role in patterning the cell as it does in the *Drosophila melanogaster* embryo. In the lab, we have recently developed an RNAi methodology for Stentor [36] that will allow us to functionally test the role of any genes that appear to be specifically induced during regeneration.

We expect that our molecular studies of regeneration and re-patterning in Stentor will reveal fundamental principles of how cells generate and regulate morphology, a general phenomenon relevant to the survival of all living systems. Cancer cells, for example, are marked by their loss of subcellular organization and recent studies have linked pathways that regulate polarity to those that suppress tumors [37]. How an individual cell establishes and maintains its subcellular organization is therefore a vital area of study in the initiation of tumorigenesis. Additionally, these studies could inform future technology development ranging from novel regenerative therapies that reactivate pathways in damaged cells to the creation of self-healing cellular robots.

## Life with 16,000 chromosomes: *Oxytricha* as a model system to study genome biology, epigenetic inheritance, and somatic differentiation

### Laura F. Landweber

The unicellular eukaryote *Oxytricha* with its extreme genomic architecture, provides a model system for many studies, including chromosome biology, post-zygotic development, epigenetics, and genome rearrangement. *Oxytricha* is a ciliated protist, and like other ciliate genera, including *Stentor* (see the preceding section from Sood and Marshall in this Forum) and the classic models *Tetrahymena* and *Paramecium, Oxytricha* shares the feature of nuclear dimorphism—the coexistence of two types of nuclei in one cell. The archival micronucleus is mostly transcriptionally silent but houses the complete diploid germline genome, which is large at over 500 Mb on at least 75 chromosomes [38], and it produces haploid micronuclei for cell mating. The second type of nucleus, the somatic macronucleus, is a highly differentiated organelle devoted to gene expression. It develops from a copy of the new zygotic micronucleus after mating. Hence, nuclear differentiation in *Oxytricha* offers a microcosm of animal development in a unicellular model, as though development progresses to a sophisticated two-cell stage, with differentiated germline and soma, but without cell division. This results in a single protist cell with multiple nuclei. Additionally, some ciliate cells contain more than one nucleus of either type.

**Fig. 3:**
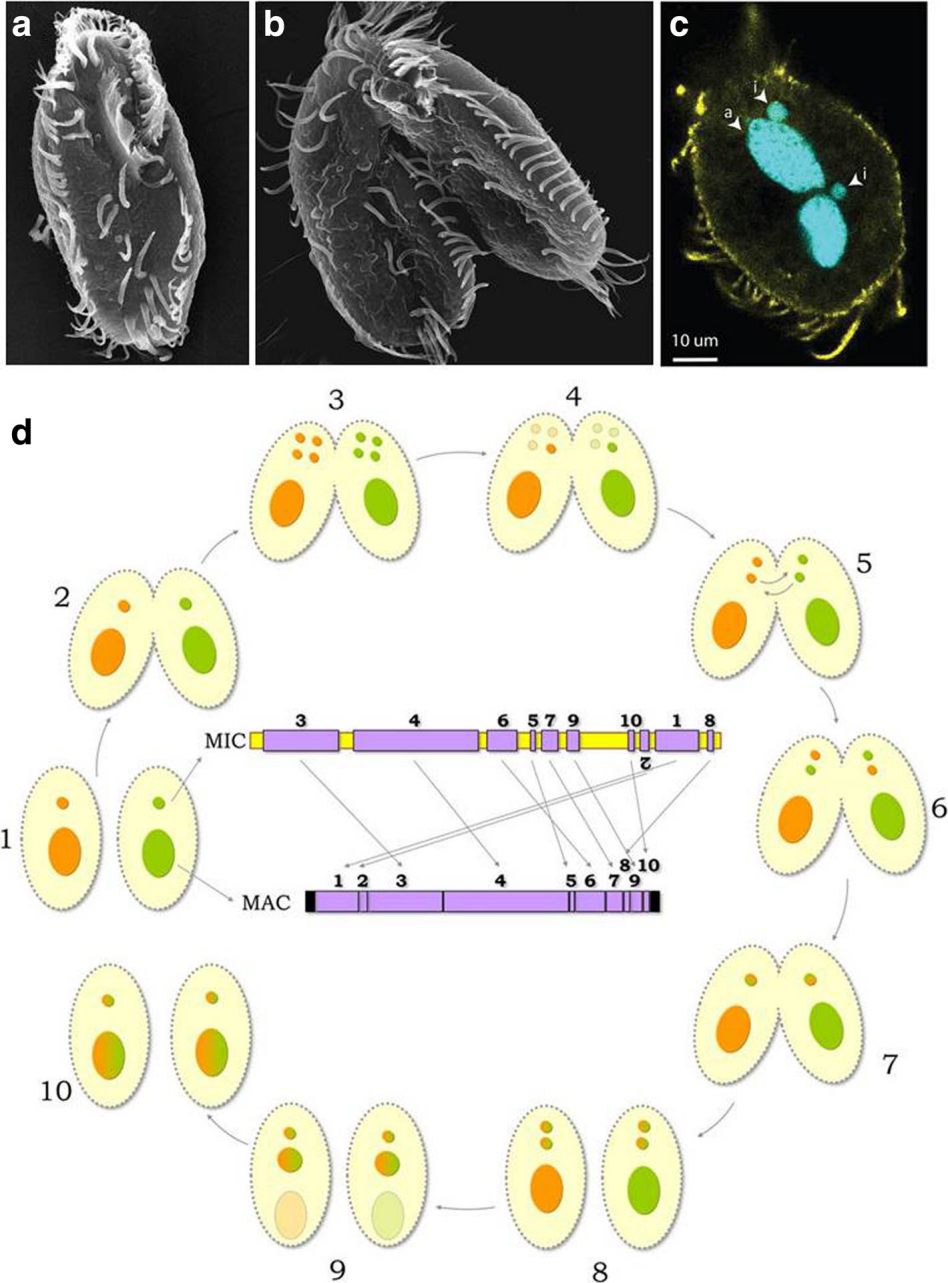
*Oxytricha* as a model system to study genome biology, epigenetic inheritance, and somatic differentiation. **a**-**c** Single (**a**,**c**) and mating (**b**) *Oxytricha* cells. Blue indicates DNA; Yellow is tubulin, highlighting the cilia. i  =  micronucleus, a  =  macronucleus. Image in (**a**) courtesy of National Human Genome Research Institute, (**b**) courtesy of Robert Hammersmith, Ball State University and (**c**) courtesy of Wenwen Fang and reproduced from [325]. **d** The sexual life cycle of *Oxytricha trifallax* and rearrangement of scrambled genes*,* reproduced from [47]*.* All vegetative cells (stages *1* and *10*) contain one (bi-lobed) macronucleus (*MAC*) and two micronuclei (*MIC*). The two MIC are genetically identical, but for simplicity we show only one here. (*2*) When starved, two cells of compatible mating types partially fuse to initiate conjugation. Cell fusion occurs soon after mixing of mating types. (*3*) Both vegetative micronuclei in each partner enter meiosis I. (*4*) One product from each meiosis I is promoted to meiosis II, and one of the four meiosis II products is promoted to a post-meiotic mitosis. (*5*) The sister products of one mitosis develop into gametic nuclei: a posterior stationary and an anterior migratory nucleus. This happens in both partners, such that both cells emerge with identical zygotic genotypes after the exchanged migratory nucleus fuses with the retained stationary nucleus (*6*), resulting in (*7*) two genetically identical exconjugant cells. (*8*) The newly formed zygotic nucleus divides twice: one daughter nucleus is destroyed, two become the new micronuclei, and (*9*) the parental macronucleus in each partner cell degrades, leaving telomere-to-telomere RNA transcripts behind to guide rearrangement [44, 45]. One zygotic nucleus differentiates into the new macronucleus. This cycle takes approximately 48–60 h. Shown inside the *circle* are representative MIC and MAC versions of a scrambled gene. Retained DNA segments in *purple*; deleted DNA regions, including flanking DNA, in *yellow*; numbered segments correspond to the order in the expressed MAC version; segment 2 is inverted; telomeres are shown as *black bars* at the ends of the MAC chromosome

The process of forming a new macronucleus involves massive DNA elimination, rearrangement, and amplification [39]. Remarkably, approximately one-fifth of all *Oxytricha* gene loci are scrambled in the germline [38]. These loci require a combination of translocation and/or inversion of DNA segments, in addition to DNA deletion, to assemble the expressed macronuclear versions (Fig. 3d). The combination of removal of nearly all intergenic DNA and loss of all satellites and transposons results in a somatic genome comprising over 16,000 tiny chromosomes that average approximately 3 kb, as well as a much smaller genome size (approximately 50 Mb) [40]. Macronuclear chromosomes lack centromeres but are capped with their own telomeres and telomere binding proteins, and thus classically *Oxytricha* was one of the first model systems for studies of telomeres and their associated proteins [41, 42]. Amplification of these “nanochromosomes” to an average copy number of ~1900 in the macronucleus [39] creates a DNA-rich and physically enormous (10–30 micron) macronucleus [43] (Fig. 3c).

A phenomenal genome editor, *Oxytricha* reorganizes its zygotic genome by stitching together over 200,000 germline DNA segments, requiring a nearly equal number of programmed DNA breakage and joining events [38]. These are accompanied by RNA-guided DNA repair [44]. Noncoding RNA molecules are at the heart of orchestrating all these complex events, with long, noncoding, maternal RNA transcripts of the previous generation’s MAC genome supplying templates for chromosome rearrangements [44, 45] and small RNAs marking the specific germline regions to be retained in the new somatic genome [46]. Therefore, *Oxytricha* provides a paragon for studies of DNA and chromosome dynamics, noncoding RNA-chromosome interactions, DNA breakage, recombination, and repair, and transposon participation [47]. The much reduced size of *Oxytricha*’s somatic nanochromosomes also makes them a unique platform for basic studies of chromatin biology (Beh *et al*., unpublished data) as well as gene regulation, genome annotation, and gene discovery [48].

The cytoplasmic mixing that occurs during mating (Fig. 3d), coupled to the fact that the cytoplasm and cell surface material of exconjugant cells explicitly derive from the parental cells, make ciliates excellent model organisms to study epigenetic inheritance (reviewed in [49]). RNA molecules are among the contents that can be directly passed on from parent to exconjugant daughter cell, and RNA-mediated transgenerational inheritance has been demonstrated via injection of foreign long or small RNAs that reprogram genome rearrangement pathways [44, 46]. These approaches for RNA-guided gene editing, facilitated by the natural machinery in the cell, also provide tools for creating somatic gene knockouts or fusion genes [50]. For example, the programmed retention of short genomic regions that interrupt reading frames [46] can introduce premature stop codons and lead to the construction of laboratory strains (that can be stored as frozen cysts) with an inability to express a gene that is normally found in the macronucleus. Additional tool development is underway and still more is needed, for example, to permit parallel screens, but *Oxytricha* is emerging as a powerful and unique model system to probe features of complex eukaryotic cells and chromatin within the confines of a single cell.

## *Naegleria gruberi*: one cell with two extreme forms of motility

### Lillian Fritz-Laylin

**Fig. 4:**
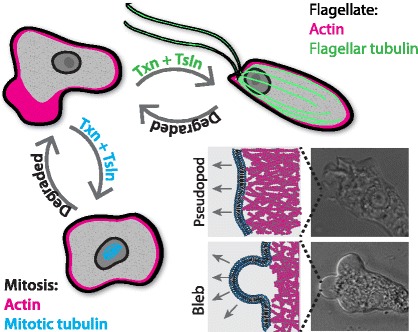
*Naegleria gruberi* cells undergo a dramatic transformation between crawling amoebae and swimming flagellates, assembling an entire microtubule cytoskeleton along the way. The crawling amoebae (*top left*) lack cytoplasmic microtubules, but use their actin cytoskeleton (*pink*) to crawl with two types of protrusions (*insets*): actin-filled pseudopods and cytoplasm-filled spheres called blebs that appear after delamination of the cell membrane from the underlying actin cortex. Amoebae can respond to a variety of environmental signals by differentiating into a vigorously swimming flagellate (*upper right*). This process requires the transcription, translation, and assembly of an entire microtubule cytoskeleton (*green*), including tubulin. Amoebae also can undergo a closed mitosis (*lower left*), during which the nuclear envelope remains intact, isolating the spindle microtubules (*blue*) from the cytoplasm. Mitotic microtubules are thought to be built from divergent tubulin isoforms that are expressed prior to mitosis, and then rapidly degraded after cytokinesis

Organisms from across the eukaryotic tree rely on two predominant forms of cell motility—crawling and swimming [51]. Each of these modes of locomotion arises from the basic characteristics of one of two conserved cytoskeletal systems: flagella used for cell swimming derive their power strokes from the sliding of stiff microtubules, while crawling motility is driven by the expansion and contraction of dynamic actin networks. The noninfectious *Naegleria gruberi* (Naegleria) takes on two extremely different forms during its lifecycle: an amoeba that crawls using actin, and a flagellate that swims with two flagella (Fig. 4). The rapid differentiation between these forms makes Naegleria a prime model for understanding both types of cell motility [52, 53].

Naegleria amoebae crawl and divide without any observed cytoplasmic microtubules [53, 54]. Not only does Naegleria undergo closed mitosis (the mitotic spindle is always contained within the nuclear envelope), but the barrel-shaped mitotic spindle lacks centrioles [54] and is thought to be built from divergent tubulin expressed prior to mitosis and degraded after the completion of cytokinesis [55]. Therefore, and remarkably, all cellular functions except mitosis are likely achieved without microtubules—including crawling at speeds topping 120 microns per minute [56]. This makes Naegleria the fastest crawling cell that I am aware of, at least twice as fast as fish keratocytes, *Dictyostelium discoideum*, and human neutrophils [57–59]. Chemical inhibitor data from other organisms suggest that rapid actin-based cell migration may not require microtubules [60, 61], and Naegleria provides biologically relevant corroboration of this hypothesis. Furthermore, there is a growing appreciation that there are multiple modes of cell migration, each driven by distinct molecular mechanisms [62–64]. Unpublished data clearly indicate that Naegleria, like *D. discoideum* and some human cells [65, 66], migrates both by using actin-filled pseudopods (a mode we call alpha-motility) and by blebbing (a delamination of the plasma membrane from the underlying actin cortex) (Fig. 4).

The differentiation of Naegleria from crawling amoebae to swimming flagellates involves assembling an entire microtubule cytoskeleton de novo, including two basal bodies (centrioles), the two flagella (cilia) that they pattern, and an entire cortical microtubule array [53, 67–71], as well as coordinating this new cytoskeleton with the pre-existing actin cytoskeleton. The process of differentiation includes transcribing and translating all of the microtubule cytoskeletal components—including tubulin—yet takes only 60–90 minutes [53, 67, 69, 72–74].

Differentiation is easily synchronized, with >90% of cells assembling basal bodies de novo within a 5–10-minute window [54, 67, 71]. (In contrast, mammalian cells take on the order of 24 hours to assemble centrioles de novo, typically after large experimental perturbations [75–77].) Recent evidence indicates that only one basal body is formed de novo, with a second in quick succession by mentored (previously “templated”) assembly [78]. The speed and synchrony of Naegleria differentiation makes it a useful model for studying centriole assembly, and in fact it was the organism in which de novo centriole assembly was first widely accepted [54]. Naegleria is also well suited for understanding how cells build motile flagella, and transcriptional synchrony of differentiation has been used to identify novel centriole and flagellar components widely conserved among eukaryotes, including humans [74].

Clearly, Naegleria is an organism uniquely positioned to reveal new insights into both crawling and swimming motility. A completely sequenced genome and publicly available transcriptional profiling of differentiation provide first steps toward harnessing this potential [51, 74]. The greatest roadblock remains the lack of usable molecular transformation and gene manipulation techniques, a hurdle we and others are actively attempting to overcome.

## R bodies: simple, dynamic protein lattices

### Jessica K. Polka

Long-range biological motion is typically the product of nucleotide-dependent motors. For example, actomyosin contraction, the bacterial flagellum, and intracellular transport along microtubules all rely on nucleotide-dependent processes carried out by complex assemblies of proteins that can be difficult to reconstitute and engineer. Therefore, if we wish to control biological motion for biotechnological applications (for example, in delivering therapeutic cargoes across membrane barriers), we should instead look for simpler systems.

Some force-generating biological machines are composed of dynamic lattices of proteins that amplify, through polymerization, the nanoscale conformational changes of their protomers to create large scale motion. Unrelated to one another in sequence or structure, these lattices are present in multiple domains of life. They include forisomes (biological “stop-cocks” that can expand to occlude fluid flow in the sieve tubes of plants [79, 80]), spasmonemes (the motile elements in the stalk of *Vorticella* that rapidly contract to withdraw the ciliate to its attachment site [81]), and R bodies (coiled structures formed in the cytoplasm of bacteria that extend, when triggered with low pH, to break membranes). Each of these structures undergoes large scale motion without relying on nucleotide hydrolysis. Because R bodies are large, genetically simple, and chemically robust, they constitute a model system to study the mechanisms of controlled self-assembly and conformational rearrangements that drive functionally related protein machines. Furthermore, they have the potential to act as a powerful chassis for engineering actuators for a variety of biotechnology applications.

**Fig. 5:**
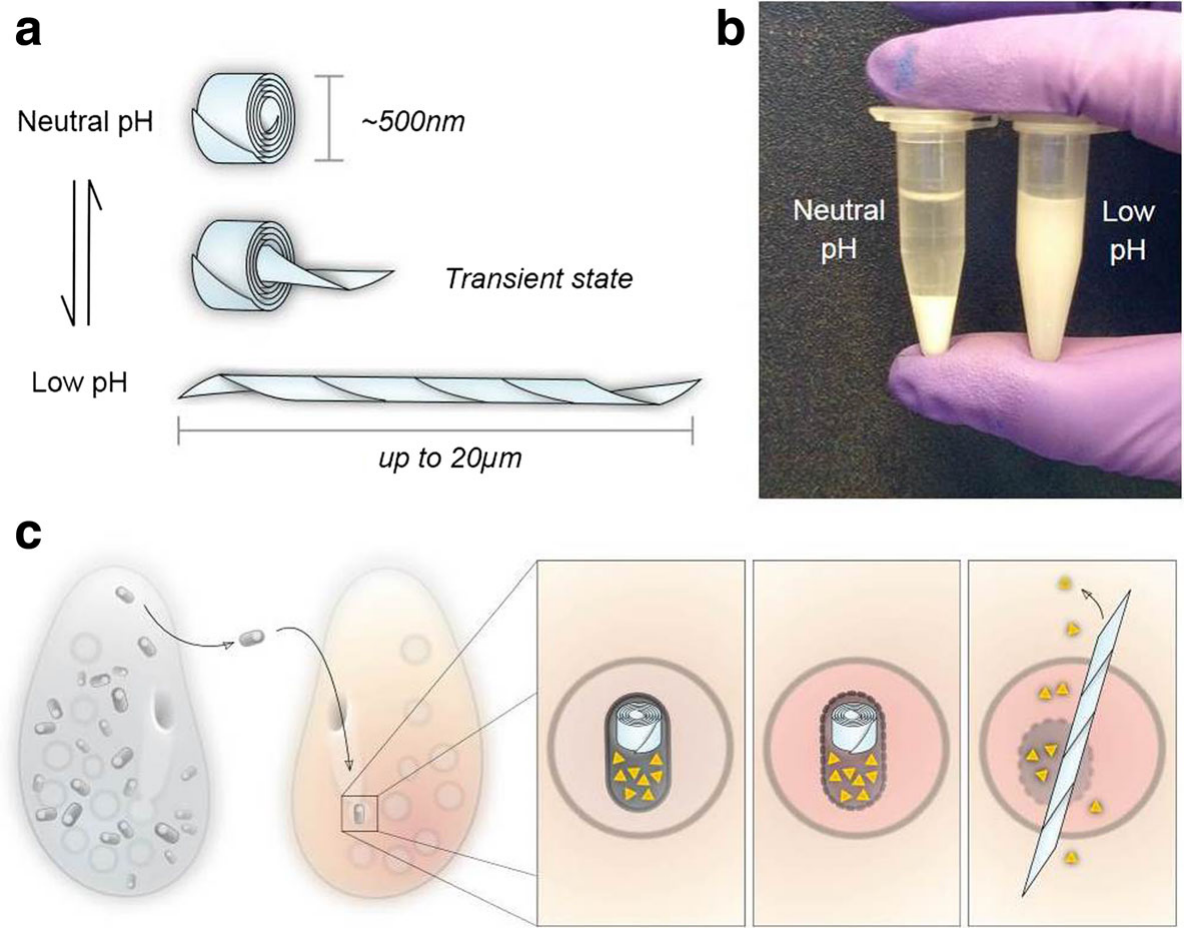
R-bodies transition between two states. **a** Type 51 R bodies reversibly transition between a rolled state at neutral pH and a tube state at low pH by the extension of the ribbon from the center of the coil. The state depicted in the middle is transient. **b** This transition is visible macroscopically; coiled R bodies sediment and extended R bodies remain in solution. **c** When an R-body-containing bacterium is ingested by a sensitive strain of paramecium after being shed by a “killer” strain, the extension of the R body is triggered by the acidic environment of the food vacuole. Extension causes the rupture of the bacterium and the food vacuole, releasing co-expressed toxins that ultimately result in the death of the sensitive paramecium. Images adapted from [89]

In nature, R bodies are coiled ribbons of protein approximately 500 nm in diameter that bacteria use to deliver toxins to eukaryotes. Upon exposure to a trigger (such as low pH) they rapidly extend in a telescopic fashion to form lancets tens of microns long. This violent extension is driven by protonation, does not require nucleotide fuel sources, and is fully reversible (Fig. 5). Sequences encoding R bodies are widespread [82], but they were first discovered in *Caedibacter taeniospiralis*, an obligate bacterial endosymbiont of *Paramecium tetraurelia* [83, 84]. Strains of the paramecium that carry *C. taeniospiralis* are capable of killing paramecium strains that do not carry the endosymbiont. Bacteria containing the R bodies are shed into the environment, where they are consumed by sensitive strains of paramecium. In the acidic environment of the paramecium phagosome, the R bodies extend to puncture the membrane and mix its contents with the cytoplasm (Fig. 5c) [85]. Feeding a diet of purified *C. taeniospiralis* is lethal to these sensitive strains, but importantly, feeding purified R bodies is not [86]. This suggests that the R body itself is not toxic; rather, it acts as a delivery system for a co-expressed toxin.

The mechanisms of R body assembly and actuation in response to low pH are largely unknown. Fortunately, R bodies are extremely simple and eminently tractable: one operon encoding four ORFS (each <120 amino acids) is sufficient for their formation in *Escherichia coli* [87, 88]. By an unknown mechanism, two of these small, nanometer-scale polypeptides can self-assemble into an organized structure many orders of magnitude larger than their individual size [87]. This process is representative of a broad biological challenge facing all cells: producing long-range order from components that interact at short length scales. By understanding the assembly processes, we may enable the production of actuators of specified mechanical properties and materials with tunable geometries.

These assembled R bodies are resistant to sonication, detergents, and diverse buffer conditions, making them stable and robust force-generating machines that could perform work in a variety of micron-scale devices. At the same time, their behavior can be probed with a simple visual assay: under appropriate buffer conditions, contracted R bodies sediment, while extended R bodies remain in solution [89]. This change can be easily seen with the naked eye as well as measured quantitatively by absorbance in a plate reader (Fig. 5b). This assay enables R bodies to be studied in a high-throughput fashion and enabled the identification of mutant R bodies that can transition at lower or higher pH than wild type [89].

R bodies’ amenability to engineering suggests that they could be used to deliver biologically active payloads across biological barriers. For example, R bodies could be conjugated to cargo such as DNA, siRNA, labeled proteins, and other chemicals. This strategy could also be used to transform recalcitrant cell types or to deliver high-value cargoes with improved efficiency.

## The awesome power of comparative fission yeast genetics

### Snezhana Oliferenko

Working on a “non-model” organism can be exceptionally rewarding because of the promise of new biology, new insights into old problems, and a whole set of new questions to solve. It might be especially tempting to venture to understudied branches of the evolutionary tree to capture the widest possible range of biological diversity. Yet, based on our experience studying mitotic division in two fission yeasts, *Schizosaccharomyces pombe* and *Schizosaccharomyces japonicus*, I want to make a case for exploring the cell biology of closely related species. Such a comparative approach is complementary to the development of new “stand-alone” systems discussed elsewhere in this Forum and I would like to argue that it can be particularly powerful if one of the two species is an established model organism.

Eukaryotes have evolved a staggering variety of mitotic mechanisms. Different species and even different cell types within the same organism may take various routes to mitotic spindle assembly [90], nuclear envelope (NE) remodeling [91], and cytokinesis [92]. For example, all dividing eukaryotic cells must remodel the nuclear envelope (NE) to allow chromosome segregation and formation of the daughter nuclei. This invariably involves major rearrangements of the NE–endoplasmic reticulum system coordinated with mitotic spindle dynamics. However, the strategies used to achieve the net result vary from fission of a seemingly intact mother NE into two daughters (“closed” mitosis) to a virtual loss of NE identity in prophase followed by its reassembly around the segregated genomes (“open” mitosis) and several strategies in between [91]. Although work by many groups provided detailed insights into the mechanisms underlying NE remodeling in a number of organisms [93, 94], we understand very little about how these circuitries evolve. Investigating this process in closely related experimentally tractable systems may explain how variation arises in evolution, probe how mitotic nuclear dynamics intersects with the rest of cellular physiology, and inform our understanding of basic NE biology and nuclear origins.

**Fig. 6:**
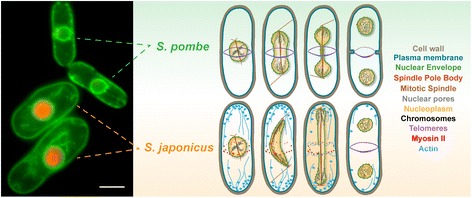
*Schizosaccharomyces japonicus* and *Schizosaccharomyces pombe* exhibit divergent mitotic programs. *Left*: Live *S. pombe* and *S. japonicus* cells expressing the endoplasmic reticulum marker GFP-ADEL and the nucleoplasmic protein Nhp6-mCherry. Note a considerably larger cell size in *S. japonicus. Scale bar* 5 μm. *Right*: Schematic representation of mitotic division in the two sister species. Adapted from [108], Current Opinion in Microbiology, Vol 28, Gu, Y. and Oliferenko, S., Comparative biology of cell division in the fission yeast clade, p.18-25, Copyright (2015), with permission from Elsevier

Fission yeasts are a small clade at the base of the Ascomycete phylogenetic tree with overall conservation of gene content and gene structure between the four species [95]. *S. japonicus* forms an early diverging branch within the clade. Strikingly, *S. pombe*, a widely used model organism, undergoes closed mitosis but *S. japonicus* breaks the nuclear membrane during anaphase [96, 97] (Fig. 6). We linked this divergence to a simple scaling argument—since nuclei maintain constant volume throughout closed division, cells must increase the nuclear surface area to form two daughter nuclei from one. It turns out that *S. japonicus* does not expand the NE during mitosis, unlike *S. pombe*, and, therefore, must break it to allow chromosome partitioning [96]. Further work showed that divergent regulation of phospholipid biosynthesis in the two yeasts through the phosphatidic acid phosphatase Lipin supports the differences in mitotic NE surface area control [98]. These observations may be a starting point in linking the underlying metabolic properties of the cell to the emergence of a particular mode of mitosis.

Another important point of divergence between the two sister species relates to differences in regulating chromatin–NE interactions during mitosis, with unexpected links to nucleolar dynamics. We have shown that although chromosomes must detach from the NE for the duration of mitosis in organisms with closed nuclear division [99, 100], *S. japonicus* has evolved an anaphase-specific mechanism supporting association between the nuclear pore complexes (NPCs) and chromatin [101]. These interactions executed by the inner nuclear membrane protein of the LEM domain family Man1 ensure equal partitioning of the nuclear membrane and efficient inheritance of the NPCs by the daughter nuclei, which essentially co-partition with segregating chromosomes (Fig. 6). It remains to be seen if variations on this mechanism function in other cell types with relatively early NE reassembly, for example, during karyomere formation in embryonic divisions in some animals [102, 103]. Yet another LEM domain protein, Lem2, functions in supporting timely NE breakage and reformation in *S. japonicus* [96]. Thus, this organism can be used as a simple model to elucidate the poorly understood molecular mechanisms responsible for functions of the evolutionarily conserved LEM proteins in maintaining nuclear structure and integrity across eukaryotes [104–106]. Perhaps more surprisingly, in *S. japonicus* chromatin–NE interactions appear to promote disassembly of the nucleolus that takes place in cells where NE integrity is lost during mitosis but not in organisms with closed nuclear division [101].

The examples above illustrate how comparing related organisms may illuminate evolutionary innovations required for attaining specific functions or identify conserved elements obscured by grossly different molecular toolkits of distant species. Knowing one of the model systems well—in our case, *S. pombe*—allows for an easier transition to a related organism, in terms of both recognizing interesting phenotypes and adapting existing technical tools. Another important advantage of working in closely related systems is the relative ease of pinpointing the divergent nodes in otherwise conserved networks supporting cell biological processes, and retroengineering the processes with novel properties in the sister species. We have been using the latter approach in our studies of mitotic NE dynamics but also to investigate division plane positioning in the two yeasts. Cells of both species divide in the middle but our studies suggest that *S. pombe*, a popular model for cytokinesis research, has evolved an unusual medial division ring assembly mechanism based on neofunctionalization of one of the recently duplicated anillin paralogs [107]. Importantly, unlike *S. pombe* that assembles the actomyosin ring in metaphase and requires a mechanism preventing its precocious constriction, *S. japonicus* initiates ring assembly only at mitotic exit, similarly to animal cells [107, 108] (Fig. 6). In general, the metazoan-like properties of *S. japonicus* division ring assembly combined with mitotic NE breakdown make it an attractive new model for studying regulation and mechanisms of cytokinesis [109].

The salient differences in cell biology between the two species outlined above are likely just the tip of the iceberg. *S. japonicus* can be used as a valuable system on its own to study phenomena not apparent in the established yeast models. Importantly, it has all the advantages of the simple experimental system, including straightforward culturing, short cell cycle, and the ease of genetic manipulations—the latter owing largely to Hironori Niki whose group developed *S. japonicus* genetic tools [110, 111] and Nick Rhind who spearheaded the fission yeast clade genomes project. Beyond its utility studying mitotic NE dynamics and other aspects of mitotic division, *S. japonicus* could become a great system for investigating the cell biology of hyphal transition [112, 113], energy metabolism [114], and centromere biology [95, 115]. Yet, it is capitalizing on the “experiment of Nature” and using the two sister species alongside each other that offers conceptually new possibilities in cell biology by expanding its *evolutionary* dimension.

## *Ashbya gossypii* as a model for cytoplasm organization

### Therese Gerbich and Amy Gladfelter

All cells face challenges in spatial organization of their contents. One solution used by eukaryotic cells is to create individual membrane-bound compartments for specialized cellular functions. But cells also need to be able to organize all the cytosolic spaces between these compartments so that biochemistry, signaling, and protein production can be tightly regulated. Gradients are one example of organization that is widely observed from micron-sized bacteria to developing insect embryos [116, 117]. How cytosolic patterns are established and maintained in spite of the dissipative power of diffusion is an area of active investigation in a variety of systems. However, the problem is especially striking in syncytial cells where many nuclei are enclosed in a large, single cytoplasm. Syncytia are found in diverse contexts, including human muscle and placental cells, many fungi, developing insects, and plant tissues. These special cell types face even greater challenges in organizing their cytosolic contents, making them a powerful place to study fundamental principles of cytoplasmic organization.

**Fig. 7:**
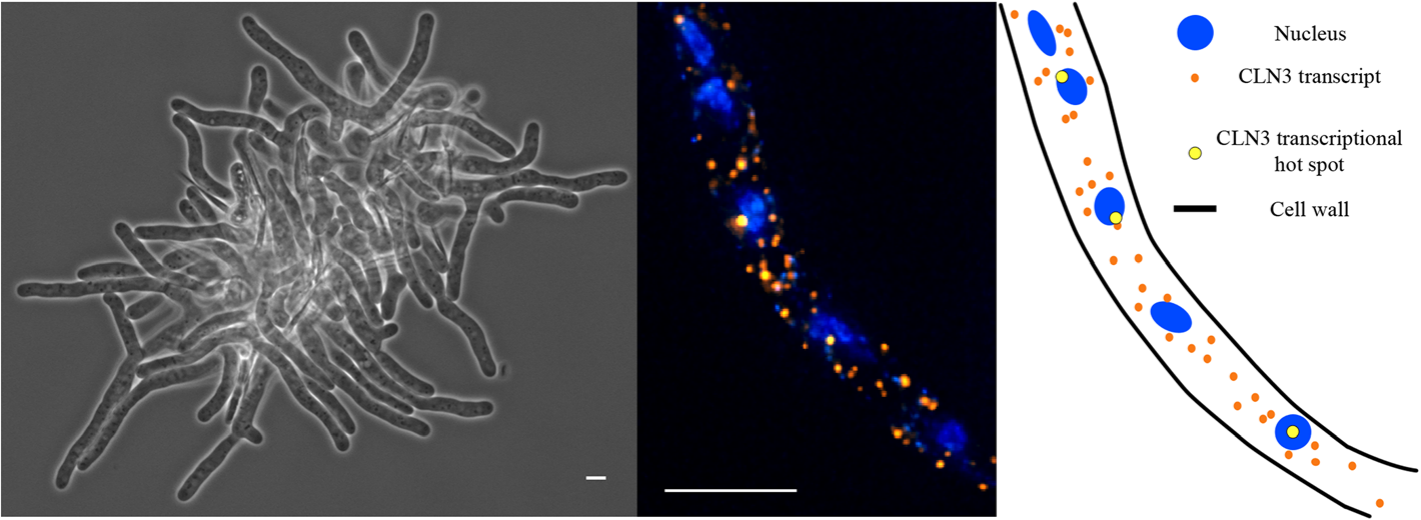
*Ashbya gossypii* as a model for cytosolic organization. *Left*: image of a growing young mycelium. *Middle*: *A.gossypii* hyphae with clustered mRNA transcripts. Asynchronously cycling nuclei are shown in *blue* and clustered cyclin transcripts in *orange. Right*: cartoon depiction of *A.gossypii* hyphae with nuclei and clustered transcripts. *Scale bars* 5 μm

A non-traditional syncytial model system that has been enormously useful for uncovering principles of cytosolic organization is the filamentous fungus *Ashbya gossypii* (Ashbya)(Fig. 7, left). Ashbya is an ascomycete that is closely related to *Saccharomyces cerevisiae*, but with a rather different lifestyle [118–120]. It has a small genome with ~4000 genes, tolerates replicating plasmids, is readily transformed, and is amenable to molecular genetics via gene targeting [121–123]. It has been a valuable system for understanding highly polarized growth and nuclear movement, and is even used as an industrial producer of riboflavin. What makes the system notable for cytoplasmic organization studies is that the many nuclei in the continuous cytoplasm go through the nuclear division cycle asynchronously [124]. This is remarkable because one would expect that all nuclei would go through the cell cycle together, as global levels of each cyclin protein rise and fall in the common cytoplasm. In studying this paradoxical cell cycle, new modes of cytosolic organization have been revealed.

Ashbya nuclei create local zones within the cytoplasm to insulate neighboring nuclei from one another so that their division cycles don’t entrain. One way these territories of cytosol form is through an RNA-binding protein that self-associates and positions mRNA transcripts of a G1 cyclin near nuclei [125] (Fig. 7, middle and left). The protein contains a large polyQ tract that enables it to form phase-transitioned assemblies that then trap cyclin transcripts in the vicinity of nuclei. If this protein can no longer undergo a phase transition and position cyclin transcripts, nuclei in the shared cytoplasm divide more synchronously [125, 126]. While the ability of proteins to undergo liquid phase transitions in vivo and in vitro had been observed previously, studies in Ashbya are one of the best connections of a biological function (positioning of cyclin transcripts to establish and maintain nuclear asynchrony) to regulated protein phase transition.

Cytosolic compartments are not just important for nuclear cycling in Ashbya but also in cell polarity. Ashbya grows exclusively in a polarized manner at cell tips such that many polarity axes coexist in a shared cytoplasm and new growth sites have to be established throughout the cell. The same RNA-binding protein that acts near nuclei forms physically distinct liquid compartments at incipient and established growth sites. These liquid droplets are important for positioning RNAs involved in polarized growth and potentially locally regulating their translation [127]. Future work in this organism will be important for understanding how different liquid compartments form, coexist, and function within a shared cytoplasm. By taking advantage of the special features that the biology of these cells offer, study of Ashbya can identify mechanisms of cytoplasmic organization relevant to all eukaryotes. A key lesson from this and all unconventional systems is that it is important to embrace biological paradoxes and try to figure them out. We have only just begun to tap into understanding the diverse ways cells and tissues solve the problems of staying alive.

## *Volvox*: revealing the origins of multicellularity and germ–soma division of labor

### James Umen

**Fig. 8:**
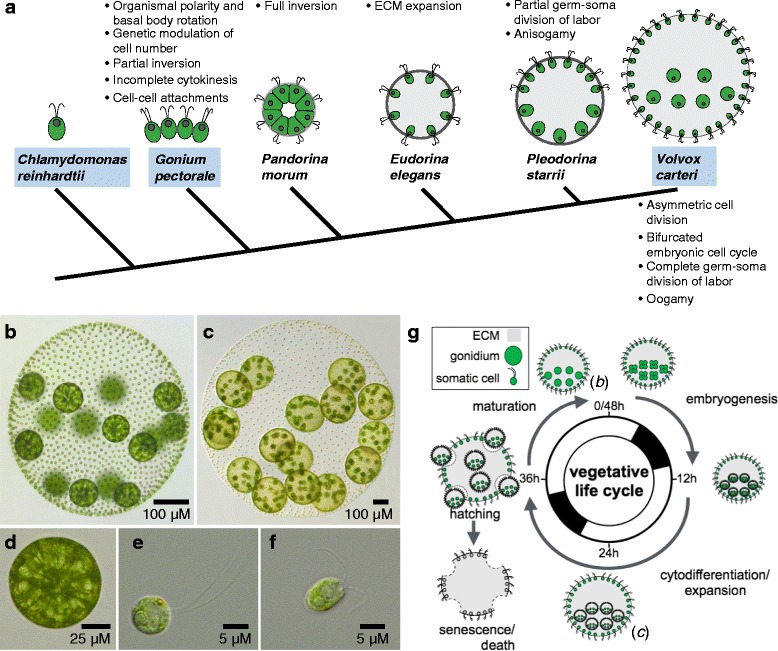
*Volvox* and volvocine algae. **a** Cladogram of selected volvocine species shown in cartoon form with successive cellular and developmental innovations indicated by bulleted descriptions above or below the node in which they arose. Species with published sequenced genomes have names in *blue-shaded boxes*. **b**–**e** Light micrographs of vegetative phase *Volvox carteri* (Volvox) showing a mature pre-cleavage stage adult (**b**); a mother spheroid with juveniles (**c**); and an isolated gonidium (**d**) or somatic cell (**e**) from a mature pre-cleavage adult spheroid. **f** Light micrograph of a *Chlamydomonas reinhardtii* cell. **g** Schematic of the Volvox vegetative life cycle synchronized to a 48-h diurnal cycle. A *boxed key* showing cell types and extracellular matrix (*ECM*) is in the *upper left*. Development starts with mature pre-cleavage adults (~11:00 on diagram) and proceeds clock-wise through embryogenesis, cyto-differentiation of germ cells (gonidia) and somatic cells in juveniles, hatching of juveniles, and maturation to become the next generation of adults. After hatching the ECM and parental somatic cells of the previous generation are discarded. The cartooned stages corresponding to light micrographs in panels **b** and **c** are labeled

Multicellularity evolving from unicellular ancestors is considered one of the major evolutionary transitions [128], with at least two dozen independent occurrences among five major eukaryotic super-clades [129–131]. Approaches aimed at understanding the origins of multicellularity, particularly for plants (embryophytes) and animals (metazoans), are challenged by the difficulties associated with reconstructing ancient events based on deeply divergent extant multicellular and unicellular lineages. *Volvox* and its close relatives (the volvocine green algae) are an alternative model for investigating multicellularity, including the early origins of traits such as cell adhesion and intercellular connections, cell-type differentiation with dedicated germ cells and terminally differentiated somatic cells, asymmetric cell divisions, morphogenetic patterning, and sexual dimorphism—all of which are found in more complex multicellular taxa. What differentiates volvocine algae from other taxa and makes them a unique model is their simplicity and their relatively recent transition to multicellularity, with several well-characterized genera that capture successive increases in morphological complexity [132, 133] (Fig. 8a). Conveniently, a close relative of all multicellular volvocine algae is the well-studied unicellular model organism *Chlamydomonas reinhardtii* (Chlamydomonas) [134, 135] that serves as an outgroup and a proxy for the ancestral state of the lineage.

*Volvox carteri* (Volvox) has been used as a “non-mainstream” model for development for several decades [136, 137], and belongs to a genus with a distinguished history that dates back to some of the earliest recorded light microscopic observations that were made by van Leeuwenhoek [138]. Vegetatively propagated Volvox individuals have a spheroidal shape and exhibit a streamlined body plan composed of just two cell types: ~2000 small terminally differentiated somatic cells arranged on the spheroid exterior with flagella oriented outward to provide motility, and ~16 large reproductive cells called gonidia located on the interior (Fig. 8b–e). All cells are embedded within an extensive clear extracellular matrix that occupies ~99% of the spheroid volume. Volvox somatic cells are similar in size and overall structure to Chlamydomonas cells (Fig. 8f), though they possess several unique derived features that distinguish them from Chlamydomonas cells in form and function [139] (Fig. 8a).

The appeal of Volvox as a model for investigating the evolutionary and mechanistic bases of multicellularity derives not just from the potential to build on several decades of detailed developmental and genetic studies but also from increasing information on related genera whose genome sequences are enabling the history of developmental innovations and their genetic origins to be reconstructed [140–142]. Intriguingly, all of the developmental regulators identified so far in Volvox (as yet only a handful) have either Chlamydomonas orthologs or are members of protein families whose origins can be traced to related families in Chlamydomonas [132]. In some cases orthologs are interchangeable between the two species, raising unanticipated questions about ancestral gene function when the trait governed by the Volvox gene has no obvious parallel in Chlamydomonas (for example, tissue morphogenesis or asymmetric cell division [143]).

As with any experimental system, the questions one can ask are dictated by available tools and resources, some of which have been reviewed recently [135]. The focus here is on Volvox, but it should be understood that Chlamydomonas has available an even more extensive molecular genetic toolkit, making it an ideal partner species for integrated and comparative cellular, developmental, and evolutionary studies. Three volvocine genomes are now published and publicly accessible—those of *Volvox carteri*, *Chlamydomonas reinhardtii*, and *Gonium pectorale* [140–142]. All three haploid genomes are similar in size (120–150 Mb) and have roughly similar gene contents with an extensive degree of 1:1:1 protein-coding-gene orthology. Several more genome sequences from species belonging to other volvocine clades are forthcoming. All volvocine algae can be propagated vegetatively (that is, mitotically) as diagrammed in Fig. 8g for Volvox, but also have facultative, inducible sexual cycles that allow mutants to be isolated and subjected to classic genetic analyses [144, 145]. Transposon tagging was developed to bypass the need for crossing which can be tricky [146–148], but there are newer isolates of Volvox that perform well in crosses [149], and in my opinion classic genetic approaches such as UV or chemical mutagenesis followed by screening, outcrossing, and whole genome re-sequencing will be the preferred way to characterize and identify mutants going forward. Volvox is transformable with exogenous DNA that integrates randomly into its haploid genome, and a variety of transgenes have been expressed including fluorescently tagged proteins, antibiotic resistance markers, and endogenous genes [150–154]. Hairpin and antisense-based gene expression knockdowns can also be done, making reverse genetics feasible [149, 155, 156]. While CRISPR-Cas9 editing has not been reported yet for Volvox, it has been successful in Chlamydomonas and could be developed for other volvocine species [157, 158].

As a developmental system Volvox has some appealing features, including organismal size and clarity that make it well suited to live-cell 3D imaging methods, including selective plane illumination microscopy (SPIM) [159]. The chlorophyll and other pigments that are in Volvox cells can interfere with live-cell fluorescence detection methods just as in plants, but more discriminating confocal microscopy technology and sensitive detection systems have helped to mitigate this issue [160]. Vegetative Volvox is easy to mass culture and will synchronize under a two-day diurnal cycle (Fig. 8g). In addition, the individual spheroids are large enough that rapid visual screens for developmental mutants can be performed using only a dissecting microscope and micropipette to pick out candidate mutants. Embryonic cleavage follows a stereotyped pattern, and the lineage relationships between cells during normal development are known; but interestingly, cell-size is the ultimate determinant of germ–soma differentiation for post-embryonic cells [161]. Many fascinating and potentially valuable developmental mutants of Volvox that affect specific multicellular and developmental traits have been described [137], and some causative genes have been identified [132], but most mutants are no longer in culture (as yet there is not a routine way to freeze Volvox cultures, though there has been some success reported [162]).

The pieces are in place to implement a promising approach for investigating multicellular innovations and their origins in Volvox using a combination of forward and reverse genetics, and making use of Chlamydomonas and other volvocine species to interrogate ancestral gene functions and origins. Descriptions of previously isolated mutants, including several that alter germ and somatic cell fates, provide an indication of the untapped riches of Volvox development [132], and with a sequenced genome and relatively inexpensive sequencing technology it is now possible to go from mutant phenotype to causative mutation in a matter of weeks. Once a mutant is identified and verified, its function can be studied not only in Volvox, but also in Chlamydomonas and other volvocine species where the causative gene is likely to have a 1:1 ortholog (or at least a homolog). In some cases Volvox and Chlamydomonas orthologs will be interchangeable in function, and in other cases not; but either result can be informative for understanding the relationships between ancestral and derived traits in the volvocine lineage. A similar combined comparative genomics and experimental approach for investigating evolutionary divergence of mitotic mechanisms in fission yeasts is described by Snezhana Oliferenko elsewhere in this Forum.

The approach outlined above is only one of several productive ways in which Volvox can be used to ask about the origins of multicellular trait innovations, and is meant to stimulate thinking about the new possibilities that genomics and other recent technologies add to this model system. Most importantly, the opportunities for exciting discoveries far outnumber the researchers who are currently using Volvox and its relatives. A great way to learn more about Volvox and volvocine algae and to tap into this research community is to attend a meeting [163, 164], or to visit a laboratory that uses these intriguing microcosms of multicellularity and experience first-hand their beauty and the scientific wonder they inspire.

## *Physcomitrella patens*: harnessing anatomical simplicity to investigate the cellular basis of tissue morphology

### Magdalena Bezanilla

Living organisms use their genome as a blue print to build intricately complex and beautiful structures. Within an organism, where every cell has the same blue print, simply controlling how the blueprint is read leads to the formation of different body parts. However, even single cells establish and maintain unique shapes, evidenced by the vast morphological diversity amongst unicellular organisms. In many organisms, cell shape stems from restrictions imposed by the extracellular environment. Eukaryotes control this by building a wide variety of extracellular structures. For example, animals build bones and shells, plants build polysaccharide walls, and diatoms construct silica-based frustules as described in this Forum by Russell and Theriot. Extracellular structures, which often are patterned over macroscopic scales, impose constraints on both cellular and tissue morphology. Yet, individual cells are responsible for depositing extracellular matrix. Thus, how organisms control this large-scale patterning of their extracellular matrices is an open question.

To gain insight into this question, it would be ideal to work on an organism whose body plan enables imaging of individual cells within tissues, and that builds a complex extracellular matrix in the context of a variety of tissues throughout development. Although land plants, with their polysaccharide walls and their indeterminate growth, certainly satisfy the latter criterion, access to individual cells within all tissues is challenging in the vast majority of vascular plants. In contrast, the moss *Physcomitrella patens* (Physcomitrella) satisfies both criteria. The Physcomitrella body plan is simple, with most tissues only a single cell layer thick, thereby providing an excellent system with which to dissect intracellular control in patterning of the extracellular matrix.

**Fig. 9:**
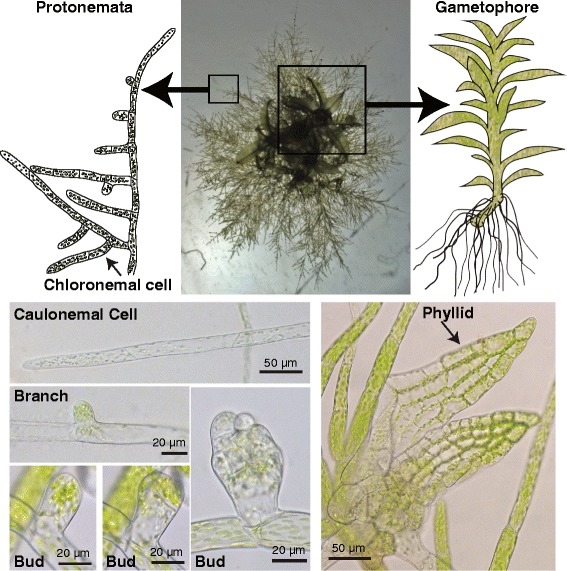
Haploid tissues of the moss *Physcomitrella patens*. A plant regenerated from protoplasts is shown in the *top center*. The *boxed regions* in this image represent the juvenile (protonemata) and adult (gametophore) tissues, which are drawn schematically on either side of the image. Images acquired from tissue grown in microfluidic devices showing a variety of cell types and tissues are shown in the *bottom row*

Physcomitrella germinates from a haploid spore, producing a linear array of cells that branch out leading to a filamentous network known as protonemata (Fig. 9). The initial cells that germinate from the spore and establish the network are chloronemal cells (Fig. 9). In protonemata, the apical cell is the stem cell, dividing leaving a subapical cell and a new apical stem cell. The filamentous network is further elaborated by branching events, whereby a subapical cell undergoes an asymmetric division generating a new apical stem cell that gives rise to a daughter filament. As the plant matures, the apical cells differentiate into a second cell type known as caulonemal cells (Fig. 9), which are characterized by faster growth and obliquely positioned cell plates. Caulonemal cells are developmentally distinct as they can grow in the absence of light whereas chloronemal cells cannot [165].

The filamentous network is the juvenile state of the plant and establishes a radial symmetry (Fig. 9). This network begins to mature into the adult plant by undergoing an additional developmental transition characterized by the emergence of buds (Fig. 9) from subapical cells. Buds represent a switch from two- to three-dimensional growth. The bud initially resembles a new branch but the apex of the cell is more rounded and the first division is oblique (Fig. 9), establishing the apical basal axis. Both daughter cells divide perpendicularly to the first oblique division [166]. This division generates the apical stem cell in the bud. The bud eventually develops into gametophores that have leaf-like structures known as phyllids (Fig. 9). Phyllids emerge regularly off the gametophore and are only a single cell layer thick.

Within the vegetative state of Physcomitrella, both juvenile and adult tissues are a single cell layer thick and thus readily accessible to microscopic observation. Furthermore, these tissues grow via distinct mechanisms. The juvenile state grows two-dimensionally by polarized secretion of extensible cell wall material to the tip of the apical stem cell, enabling turgor-driven cell expansion only at the cell apex. In contrast, the adult state switches to three-dimensional growth characterized by diffuse cell expansion. Strikingly, the gametophore is generated from a single apical stem cell [166], a dramatic simplification in comparison to seed plants, which have an apical domain known as the meristem comprised of several layers of undifferentiated stem cells.

Even with the relatively simple anatomy of Physcomitrella, continuous imaging over developmental time of events that occur in the denser regions of the filamentous network, such as caulonemal cell maturation and bud formation, has been challenging. Phyllid expansion that occurs in the air and in three dimensions was also not accessible to high-resolution imaging. Recently these limitations have been largely overcome by the ability to grow Physcomitrella in custom-made microfluidic imaging chambers for weeks [167], providing the unique opportunity to observe protonemal tissue differentiation, bud formation, and phyllid expansion at cellular and subcellular resolutions.

Another feature that makes Physcomitrella a particularly useful model system is the ability to propagate the plant vegetatively. Upon mechanical disruption, cells in the damaged tissue de-differentiate into chloronemal cells, re-establishing a new plant. This effectively enables indefinite propagation of any Physcomitrella line, which is especially useful for mutant strains with developmental defects. As an extreme example of vegetative propagation, it is possible to remove the cell wall from Physcomitrella tissue enzymatically, generating a suspension of protoplasts, which given appropriate osmotic conditions then rebuild their walls and generate a new plant resembling one germinated from spores.

Protoplasts are also easily transformable with DNA [168] opening the door to genetic manipulations. Among these is the ability to use homologous recombination for gene targeting [169–171], a feature unique to mosses amongst land plants, which has made it possible to generate lines that express native proteins fused to reporter genes [172] or fluorescent proteins expressed from their own genomic context [173]. Most recently CRISPR-Cas9-mediated gene targeting has also been shown to generate genomic lesions effectively [174, 175]. Because juvenile and adult tissues are haploid, a genomic lesion immediately results in a mutant. Additionally, RNA interference (RNAi), which can target multiple genes simultaneously, can be performed transiently [176, 177] or inducibly [178], enabling loss-of-function studies of whole gene families. Finally, since whole-genome sequencing has become routine, it is also possible to identify genomic lesions introduced by random mutagenesis [179].

The extensive genetic tool box coupled with facile imaging of single cells within the context of whole tissues uniquely positions Physcomitrella among land plants as an excellent model organism. In addition to interrogating how molecules within individual cells pattern extracellular matrix over macroscopic length scales, Physcomitrella provides the opportunity to answer key questions in plant cell and developmental biology.

## Cerebral organoids model human brain development and disease

### Madeline A. Lancaster

For centuries, the human brain has been one of the most difficult organs to study. The brain is what makes us unique, both as individuals and as a species. But for this very reason, its particular features are impossible to study in other organisms, and ethical and methodological limitations prevent us from directly studying it mechanistically. So while animal models have provided insight into what it is to be a vertebrate, a mammal, or even a primate, there still remain many questions surrounding what it is to be human. For example, while neural stem cells behave in much the same fashion in all vertebrates, their neurogenic potential is greatly increased in humans, giving rise to over a thousand times the number of neurons in a mouse brain [180], and a brain that is over three times larger than our closest relatives, chimpanzees and bonobos [181]. Furthermore, there are important differences in cytoarchitectonic organization, such as the presence of grey matter minicolumns [181] and numerous unique interneuron populations in the cortex [182, 183], and overall denser, more complex dendritic trees and spines [184].

Because of these unique features, it has proven difficult to recapitulate many human neurological disorders accurately in mouse models. For example, primary microcephaly (small head size) in humans is caused by homozygous null mutations in any of a number of centrosomal or DNA repair genes, yet when these mutations have been introduced in mice, the effect on brain size is minimal [185]. Likewise, mouse models of human mutations seen in neurodegenerative conditions fail to display the full range of defects, such as both plaques and tangles seen in brains of patients with Alzheimer’s disease [186]. These are just a couple of the numerous failures to model human neurological conditions in traditional animal models, which unfortunately has led to a drying up of the drug pipeline in this area, and a lack of further interest on the part of the pharmaceutical industry [187].

Recently, neuroscientists have turned their attention in vitro with the hope that human features might be modeled using human neural cells. However, until very recently, in vitro meant a disorganized layer of cells grown in 2D, hardly capable of being considered a model of any developing organ. Then, in 2001, Zhang *et al*. established the first so-called neural rosettes [188], which modeled with remarkable fidelity the epithelial arrangement of neural stem cells and the formation of neural tube-like lumens. Over the next 10 years, improvements were made in the reproducibility and efficiency of formation of neural rosettes [189, 190], and in 2008, Eiraku *et al*. published the SFEBq method [191] for generation of larger, more complex rosettes as a result of culturing in 3D before plating tissues in 2D. Building upon these studies, in 2013, we developed a completely 3D model system of human brain development: so-called cerebral organoids [192]. Because of their reliance on endogenous signals, cerebral organoids are capable of remarkable self-organization resulting in complex tissues containing a variety of interconnected brain regions. That same year, Kadoshima *et al*. established a 3D method for generation of forebrain tissues [193], and in 2015, Paşca *et al*. developed a method for generating spheroids containing cortical rosettes [194].

**Fig. 10:**
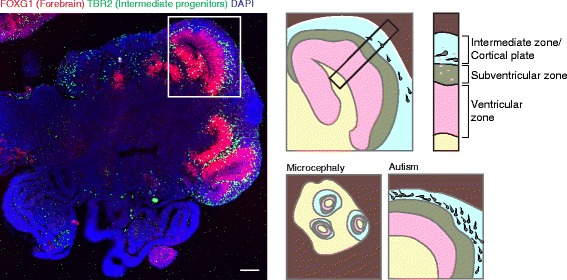
Cerebral organoids model the architecture of the developing human brain. *Left*: a section of an entire cerebral organoid stained for the forebrain marker Foxg1, the intermediate progenitor marker Tbr2, and DAPI, revealing the presence of lobules of cerebral cortex as well as other brain regions not positive for Foxg1. *Right*: a schematic of a lobule of cortex in an organoid showing the proper organization of progenitor zones: ventricular zone (VZ) where radial glial neural stem cells reside, subventricular zone (SVZ) where transit amplifying populations reside, and the intermediate zone (IZ) and cortical plate (CP) where neurons migrate to their final positions. Scattered *pink puncta* represent outer radial glia, a population abundant in human brain development but much less present in rodents, while elongated *purple neurons* represent tangentially migrating interneurons that originate outside the cortex. In the case of microcephaly (*lower left*) organoids overall are much smaller, as are progenitor zones [192], whereas organoids derived from autistic patients display increased numbers of interneurons [198]. *Scale bar* 100 μm

Overall, the methods that have arisen in the past 5 years have revealed the remarkable ability of stem cells to self-organize and form tissues reminiscent of the early developing brain. While cerebral organoids contain a variety of brain regions with remarkable complexity, spheroids generated with exogenous patterning factors and small molecules more reproducibly generate forebrain and cortical rosettes [195]. But one thing all the methods have in common is the ability to accurately model the behavior of neural stem cells and their organization into discrete progenitor zones highly reminiscent of the tissue architecture in vivo. Because of their organization, species-specific differences in tissue architecture and stem cell behavior can be detected in neural organoids (Fig. 10). For example, human cerebral organoids display large numbers of outer radial glia [192, 193], an extra population of neural stem cells that is highly abundant in the developing primate brain, but limited in mice. Furthermore, differences in both neural stem cell division dynamics and fate have recently been described between human and non-human primate organoids [196, 197].

The fact that brain organoids display human-specific features holds great promise for their use in modeling neurological disorders. Indeed, despite their very recent development, neural organoids have already been demonstrated to model features of microcephaly [192], autism [198], lissencephaly [199], and even Zika virus infection [200, 201]. A further testament to their utility is the increasing adoption of these methods in numerous independent laboratories. As with many novel technologies, widespread adoption takes time and so from the cerebral organoid paper in 2013 through 2015 only four publications made use of 3D neural organoids. But last year alone this number jumped to 19 and there is no sign of slowing in the immediate future. While it is still early days, the hope is that the application of brain organoid methodologies to the study of an increasing number of neurological syndromes will provide a treasure trove of new insight into disorders of this previously enigmatic organ.

## *Nematostella vectensis*: born to be a starlet

### Shuonan He and Matthew C. Gibson

**Fig. 11:**
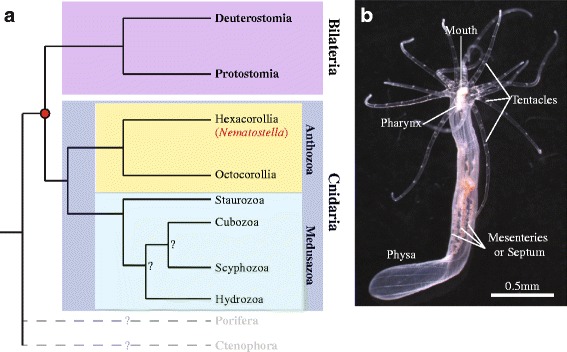
*Nematostella vectensis* phylogenetic position and juvenile morphology. **a** Metazoan phylogenic tree highlighting the position of *Nematostella*. The detailed ingroup relationships of medusozoa, as well as the position of Ctenophora and Porifera, are still uncertain, as indicated by *question marks*. **b** Morphology of a fully relaxed juvenile *Nematostella vectensis* polyp. The thickened internal foldings along the body column are called mesenteries. These delicate structures contain digestive glands, and retractor muscles as well as gonads. The *reddish coloration* of mesenteries is due to feeding of *Artemia nauplii* under laboratory conditions. Tree in **a** adapted from [209, 326, 327]: Bridge, D; Cunningham, C W, Class-level relationships in the phylum Cnidaria: molecular and morphological evidence, Molecular Biology and Evolution, 1995, Volume 12, Issue 4, p.679-89, by permission of Oxford University Press and Society for Molecular Biology and Evolution

Cnidarians have long attracted attention from biologists and it is easy to see why. From Abraham Trembley’s classic illustrations of regenerating hydra to Ernst Haeckel’s vivid depiction of discomedusae and sea anemones in *Art Forms in Nature*, these delicate creatures exhibit an exotic beauty [202, 203]. For contemporary studies of evolutionary cell and developmental biology, cnidarians have begun to offer much more than simple visual appeal. Widely accepted as the sister group to bilaterian animals, cnidarians possess apparent radial symmetry, lack definitive mesoderm, and have only a single opening that functions as both mouth and anus [204, 205] (Fig. 11). Beyond aesthetic intrigue, these morphological distinctions indicate key evolutionary transitions in the bilaterian lineage after the split of both phyla from their common ancestor, making cnidarian biology central to our understanding of animal evolution. Nevertheless, more than 250 years after Trembley’s pioneering work, we still know surprisingly little about the molecular mechanisms that dictate the distinguishing morphological features of cnidarians. One major obstacle has been the absence of a singular cnidarian species that is equally tractable for developmental, cellular, and genomic analysis.

Addressing this issue, the starlet sea anemone *Nematostella venctensis* (Nematostella) has emerged at the forefront of cnidarian model systems with the potential to serve broad research interests.

Nematostella is an estuarine, burrowing sea anemone, first described and named by Thomas Stephenson in 1935 [206]. In the wild, they can be found in brackish ponds or marshes along the coast with recorded salinities ranging from 8.96 to 51.54% and water temperatures from −1 to 28 °C [207, 208]. Adaptation to such an ever-changing habitat might explain why Nematostella is exceptionally easy to culture in the laboratory compared with most other cnidarian species. Nematostella belongs to the class Anthozoa, which consists of corals, sea anemones, and sea pens. Phylogenic analysis based on morphology, rRNA, 18S rDNA, and mtDNA data placed Anthozoa at a more basal position within the cnidarian phylum [204, 209–212]. Indeed, it has been proposed that the Anthozoan life history (with only a sessile polypoid adult form) represents the ancestral state of cnidarians [213–215]. If the polyp-first hypothesis is correct, comparative studies using Nematostella are ideal for reconstructing morphological traits of the putative bilateria–cnidaria common ancestor.

Nematostella is a dioecious species. Although sexual plasticity has been reported in other Anthozoans, this phenomenon has not been observed in Nematostella [216, 217]. In the lab, spawning can be induced easily by subjecting sexually mature animals to a combination of light and heat shock [208, 218, 219]. During spawning, females produce gelatinous egg masses, each containing hundreds of eggs, while males release sperm directly into the water. This highly controllable spawning process enables access to large quantities of synchronized developing embryos that are amenable for further experimental manipulations. Nematostella has a simple Anthozoan life history with no medusa stage. The fertilized egg undergoes a series of “chaotic” cleavages, and quickly forms a well-organized single epithelial layer. Epithelial polarity is established in early cleavage stages, providing a perfect system to study epithelial formation, growth, and morphogenesis during early embryogenesis. Embryos gastrulate around 20 hours post fertilization, developing only two germ layers, the ectoderm and the endoderm (also referred to as the entoderm or endomesoderm). Within 48 hours post-fertilization, the embryo develops into a fully ciliated, free-swimming planula larva and starts to escape from its surrounding gelatin matrix. By day seven, elongated planulae settle down and metamorphose into polyps bearing four tentacles [220, 221]. Under optimal conditions, it takes 2 to 3 months for a juvenile polyp to reach sexual maturity. Once sexually mature, spawning can be induced every 2 to 3 weeks year round without damaging the animals. Nematostella can also undergo asexual reproduction, which usually occurs via transverse fission through the body column, and can be triggered by extensive feeding, even prior to sexual maturation [222]. Interestingly, the life span of Nematostella remains undetermined as it apparently exceeds the “life span” of a PhD student or postdoc.

Nematostella was the first cnidarian to have its whole genome sequenced. The high-quality genome sequence revealed the presence of the majority of the gene repertoire for bilateria development and biochemical processes in the eumetazoan ancestor [223]. More strikingly, at the genomic level, vertebrates share more similarities with Nematostella than with ecdysozoans (including, for example, fruit flies and nematodes) [223–226]. Despite the notable conservation of intragenic sequence and gene structures, the conservation of function as well as regulation of these genes remain poorly explored. Fortunately, a rapidly expanding Nematostella toolkit will fulfill this purpose. Morpholino and mRNA delivery via microinjection has proven to be a powerful approach to manipulate gene expression level [227]. Meganuclease-mediated transgenesis is also well established, which helped the generation of several tissue- and cell lineage-specific reporter lines [228–230]. Genomic approaches such as ChIP-seq enable the identification of potential enhancer/promoter regions for certain genes and allow a careful dissection of the gene regulatory network [231]. Most importantly, to our knowledge, Nematostella is the only cnidarian system where TALEN- and CRISPR/Cas9-mediated genome editing has been reported [232, 233]. The ability to generate knock-out as well as knock-in mutants opens up new possibilities and finally permits sophisticated genetic analysis of gene functions in a cnidarian species.

Over the past decade, studies on Nematostella have shed light on a few fundamental innovations of bilaterian evolution, including the determination of body axis [233–236], the origin of mesoderm [237, 238], and the emergence of a centralized nervous system [229, 230, 239, 240]. Through these studies, a surprising new picture is emerging of a morphologically and genomically complex eumetaozoan ancestor. Paralleled by progress in other cnidarian model systems [241–244], future research using Nematostella will provide new insights into common molecular mechanisms behind the diversity of life and promises to reshape our understanding of animal evolution.

## Water bears: evolution of body forms and survival of extremes

### Bob Goldstein

In May 1997, a new molecular phylogeny of the animals revealed that *C. elegans* and *Drosophila* were much more closely related than had been thought [245]. Previous phylogenies had placed the nematodes (which include *C. elegans*) and arthropods (which include *Drosophila*) so distantly from each other that arthropods were thought to be even more closely related to *us* than they were to nematodes. But the new work revealed that these two groups were united along with a handful of hard-to-pronounce animal phyla: onychophorans, kinorhynchs, priapulids, nematomorphs, tardigrades, and later the loriciferans as well. I thought that this branch of the tree of life would be a terrific place to look for new models for comparative biology that could take advantage of the two strong model systems nearby. I was especially interested in studying how developmental mechanisms evolved in ways that produced diverse animal forms, and I figured that *C. elegans* and *Drosophila* could be rich and ongoing sources of information for comparative studies.

**Fig. 12:**
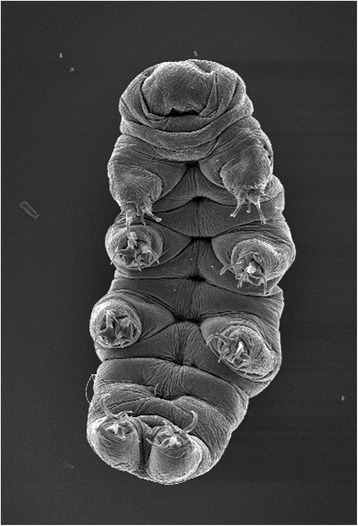
Scanning electron micrograph of the water bear *Hypsibius dujardini*. Image credit: Vicky Madden and Bob Goldstein

Tardigrades, better known as “water bears”, are eight-legged microscopic animals (Fig. 12). These animals live just about everywhere, and remarkably, they survive desiccation, so they can be found readily by placing clean biological substrates such as mosses or lichens in spring water. We were fortunate that amateur scientist Bob McNuff had been growing water bear cultures continuously in his home for two decades [246], apparently overcoming historical difficulties with keeping cultures long-term [247], and he generously shared his culture methods. And another lab had begun to collect sequence data [248]. The species we had chosen, which Roberto Bertolani kindly identified for us as *Hypsibius dujardini* [246], has a short, two-week generation time, and is easy to keep as living cultures in the lab or as frozen stocks [246].

Our initial work was necessarily descriptive. PhD student Willow Gabriel and I observed and described embryonic development and started to build a cell lineage. We found that unequal cell divisions, nuclear migrations, and cell migrations occurred in stereotyped patterns in each embryo [246]. And we began to develop tools, including methods for immunostaining and enzyme histochemical staining [246, 249]. Postdoc Jennifer Tenlen developed methods for RNA interference, which made feasible for the first time investigations into the functions of individual water bear genes [250]. We viewed these methods as forming a platform for investigating two topics: how developmental mechanisms evolve in ways that produce novel body plans, and how animals and biological materials more generally can survive in extreme environments.

Water bears are a convenient case study for asking how unique animal body plans arose because water bears share with arthropods highly modular body plans, composed of segments. This fact gave us some hope that homologous body parts could be recognized readily between water bears and organisms like *Drosophila*. Postdoc Frank Smith, who had developed *in situ* hybridization methods for water bears, has sought to understand how the compact body plan of water bears arose long ago, back when the major groups of animals had diverged. Frank found that the Hox genes that define the head segments of arthropods are expressed in the same anterior-to-posterior register in water bears—but throughout almost their entire body—leading the animals to be called at times “walking heads” [251, 252]. Water bears’ compact bodies appear to have arisen by loss of a large part of an ancestral body plan, corresponding to the entire thorax and nearly the entire abdomen of *Drosophila*. This work revealed that animal body plans can arise by loss of a large body part, and a far larger part than we had anticipated [251]. How the finer, essential details of water bear anatomy first evolved and later diversified is not yet known. Work on these questions will likely benefit from *Drosophila* and *C. elegans* as sources for candidate mechanisms.

Water bears are among just five animal clades with representatives known to survive desiccation, together with certain arthropods, rotifers, nematodes, and flatworms [253]. Among these organisms, water bears have to date survived the most remarkable environmental extremes, including freezing in liquid nitrogen in the hydrated state, freezing to within a degree of absolute zero in the dried state, and more than 4000 grays of ionizing radiation in the dried or hydrated state [254]. In September 2007, desiccated water bears were launched in a Soyuz rocket and then exposed to the vacuum of space for 10 days. Upon rehydration, animals survived and produced young that hatched at normal or nearly normal rates [255, 256]. Many of these extreme conditions should damage even what water bears and other organisms are made of—DNA, proteins, membranes—suggesting that water bears must produce protectants [254]. Postdoc Thomas Boothby sought to identify protectants, using transcriptome sequencing and RNA interference to identify essential protectants induced by extreme conditions, and then expressing the identified components in other kinds of cells to test for sufficiency to promote tolerance to extremes. This work has identified a set of water bear-specific proteins that promote desiccation tolerance [257]. Other groups have identified a water bear-specific chromatin-associated protein that can protect even human cultured cells from DNA damage [258]. Water bears may well serve as a continued source of a variety of molecules that can protect diverse molecular components against diverse kinds of extreme conditions.

Water bears have fairly complete and well-assembled genomes [258, 259]. Work using water bears would benefit tremendously at this stage from the development of methods to insert genes and edit the genome. In the meantime, there are many tools that can be applied to water bears, and other emerging models discussed in this Forum, to help unveil mechanisms of interest [260].

## Axolotls as models for regeneration

### Elly M. Tanaka

How biological systems restore missing parts is fascinating at every level: at the level of cells, cellular aggregrates, and embryonic regulation; but perhaps the most remarkable example is the regeneration of the tetrapod limb as seen in salamanders including axolotls. Upon limb severing—which, as salamanders tend to be a carnivorous sort and eat each other’s body parts, is not uncommon—the remaining cells jump into action to exquisitely replace each and every tissue type, including blood vessels, muscle, bone, nerve tracts, and skin, with the correct shape and function. How does this system work? Much of the tissue-scale logic of regeneration was worked out by capitalizing on the remarkable graftability of salamander tissues. For example, a limb blastema grafted to another body location still regenerates the limb it would have in situ, which showed us that the cells residing in the limb and tail have a memory of their position [261, 262]. Tissue-specific roles were defined by blocking regeneration through irradiation and then rescuing it with grafts of normal tissue, which, for example, gave the first indications that dermal cells play an important role in regeneration of patterned skeleton [263]. Molecular analysis proved challenging due to the need to work in adult tissue, but over time, all the required approaches to manipulate cell and molecular function have been developed, and it has been an exciting time to delve into the mechanisms of regeneration.

When choosing any model organism for research, one has to carefully consider why. I chose the axolotl as a model organism to study limb and spinal cord regeneration because it was one of the few species that was easily breedable in the laboratory, and therefore allowed for the development of transgenesis and, more recently, CRISPR-mediated gene mutation to study regeneration [264–268]. These genetic approaches are facilitated by the ability of the animals to lay up to 500 eggs per mating. Furthermore, several natural mutant strains exist, including those with absolutely no skin pigment. This allowed us to implement live imaging of fluorescent protein-expressing cells in larval axolotls to identify the cells that build the regenerate. Viral approaches to gene expression have also been very useful [269–272].

**Fig. 13:**
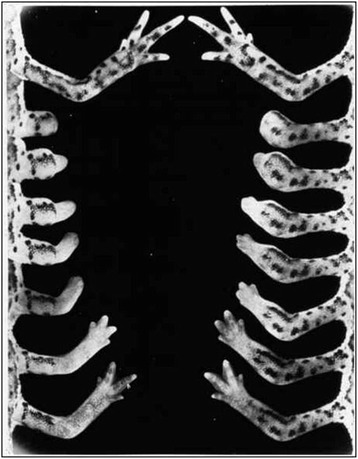
Limb regeneration in salamanders. Reprinted from [328], Elsevier Books, Richard Goss, Principles of Regeneration, Copyright (1969), with permission from Elsevier

Several other features make the axolotl advantageous for tissue imaging studies. Due to the large genome, the cells are very large and therefore can be tracked using low magnification objectives with long working distances. This tissue is quite hardy, and is highly receptive to electroporation as a means of transfection. Furthermore, being cold-blooded, the animals can be kept at a variety of temperatures, including room temperature [273]. With the development of these molecular genetic and imaging capabilities, it has been possible to pinpoint the cells that form the blastema, and to start to identify and study the cues that initiate and sustain regeneration, as well as pattern the regenerate (Fig. 13).

Axolotls are certainly not the only experimental salamander system, with work in *Cynops pyrroghaster*, *Pleurodeles Waltl*, and *Notophthalmus viridescens* each bringing a different set of opportunities to understand regeneration biology and its diversity [274–277]. We have been astonished by the divergence in the implementation of skeletal muscle dedifferentiation as found in *N. viridescens* but not in axolotls, who use muscle stem cells to regenerate their muscle tissue [278].

I think that with the new developments in genome engineering that are available, it is a wonderful time for researchers to re-assess and survey metazoans, and look to Nature for those organisms that provide amplified traits that help us to solve biological questions. The axolotl is a great example of an organism that presents unique opportunities to study biology, that has a unique set of experimental advantages, and that has recently opened up to highly molecular, mechanistic approaches. It will be exciting to define further the genetic programs that convert cells from the adult state to the regeneration state while retaining their positional memory, and to investigate the role of humoral factors, including the immune system, in this process.

## Stop the clock: the killifish model of aging in diapause

### Chi-Kuo Hu and Anne Brunet

#### Aging biology and model organisms

How do we age? This may be one of the most intriguing questions in biology. Aging is a progressive process that converts young and healthy individuals into old and decrepit ones, thereby limiting their lifespan. In nature, lifespan is an amazingly diverse trait, with maximal lifespans ranging from days in the medfly to over 500 years in clams [279]. This diversity opens up many possibilities for new model systems for aging and lifespan studies.

Much of our understanding of aging comes from studies of short-lived non-vertebrates (for example, *S. cerevisiae, C. elegans*, or *Drosophila*) [280–282]. While many key “aging genes” are evolutionarily conserved, the aging process in vertebrates is considerably more complex than in non-vertebrates. For example, non-vertebrates lack an adaptive immune system, which underlies many aspects of vertebrate aging via “immunosenescence” (gradual deterioration of the immune system during aging) [283] and “inflammaging” (chronic inflammation that occurs during aging) [284]. However, canonical vertebrate model organisms such as mice and zebrafish have relatively long lifespans (a maximum of ~4 and ~5.5 years in mice and zebrafish, respectively [285, 286]). This is a critical experimental hurdle to study vertebrate aging.

To fill this gap, we and others have developed the African turquoise killifish *Nothobranchius furzeri* as a model organism for vertebrate aging [287–294]. The turquoise killifish is the shortest-lived vertebrate that can be bred in captivity, with a maximal lifespan of 7–8 months [288, 289] (C-KH and AB, unpublished data). This is about an order of magnitude shorter than mice and zebrafish. Despite its short lifespan, the turquoise killifish recapitulates various stereotypical aging traits that have been reported in other vertebrates, including decline in normal functions and increased risk of diseases such as cancer [295–297].

#### Lifecycle of the African turquoise killifish

**Fig. 14:**
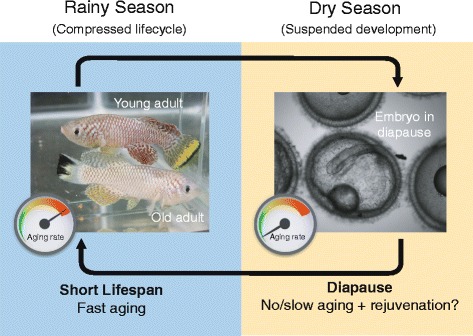
The African turquoise killifish has two distinct phases in its lifecycle. During the rainy season, the turquoise killifish has a naturally compressed lifecycle. Turquoise killifish grow fast and rapidly reach sexual maturation, characterized by bright colors in males (*Young adult*). Old fish recapitulate aging phenotypes, including loss of muscle mass, color, and tissue homeostasis (*Old adult*). Newly laid turquoise killifish embryos enter diapause to survive the upcoming drought during the dry season (embryo). The embryos can stay in diapause for many months (several times longer than the fish lifespan), raising the possibility that the damage that accumulates with time may be slowed or even reset (“rejuvenation”). The embryos then break diapause and the fish resume their compressed lifecycle during the following rainy season. Some embryos escape diapause, and it is therefore possible to study each state separately in the laboratory

The short lifespan of the turquoise killifish is likely a consequence of an evolutionary adaption to its extreme habitat. The turquoise killifish naturally lives in ephemeral ponds in southeastern Africa, which entirely dry up during the dry season. This species switches between two distinct phases (Fig. 14) [298]. The first phase takes place during the rainy season and consists of a compressed lifecycle (~40 days from embryos to embryos of the next generation), in which the turquoise killifish grows fast, reproduces fast, and, likely as a consequence of these constraints, also ages fast. The second phase takes place during the dry season and consists of a state of suspended development called diapause, which enables embryos laid during the rainy season to survive through the drought—lasting months or even years [299]. Notably, to hedge the risk, some embryos naturally skip diapause and exhibit a continuous lifecycle [300] (C-KH and AB, unpublished data). This feature allows turquoise killifish colonies to be conveniently maintained in captivity without the hurdle of diapause [294]. In captivity, both phases of the lifecycle remain unchanged, even in constant water, indicating that both the short lifespan and diapause of this species are under genetic determination.

#### Genetic and genomic resources of the African turquoise killifish

Since the initial characterization, a comprehensive toolset to study killifish has been developed. This includes a standardized strain (in this case, GRZ [301])—one of the critical features of a model organism. Several other strains are reaching the status of inbred lines (C-KH and AB, unpublished data), providing the community with additional options of genetic backgrounds, thereby minimizing the risk of strain-specific artifacts.

Great progress has also been made in developing genomic and genetic tools for this fish [294, 302–305]. We and the Platzer group have independently de novo assembled and annotated a reference genome for this fish [288, 289, 306, 307] along with numerous transcriptomic and epigenomic datasets [308–313]. An integrative reference genome generated by the NCBI pipeline with publicly available omics resources is available online (NCBI Genome ID 2642 [314]), and the effort to integrate the two genomes is currently ongoing. More recently, transgenesis and highly efficient CRISPR-Cas9 genome editing have been developed, with the ability to generate knockin or knockout lines in just 2 to 3 months [293, 302].

#### Short lifespan and compressed lifecycle: unique features for lifespan studies

A key hurdle for all lifespan studies is the need to control environmental variables throughout the whole lifespan of the organism. Longitudinal control of environmental variables can be challenging in canonical vertebrate model organisms such as mice, due to their longer lifespan, but it is easier in the turquoise killifish. In addition, relatively high throughput studies (for example, to test several genes or compounds simultaneously) are more feasible in the killifish, due to the low cost of maintaining a large cohort of animals. Finally, systematic longitudinal studies to predict individual lifespan trajectories are also more practical in the turquoise killifish than in longer-lived species [313].

#### Diapause: another key feature of the African killifish

In addition to the fast aging process of the turquoise killifish, which has clear values for vertebrate aging studies (reviewed extensively elsewhere [302]), the diapause phase of this fish provides a unique foray into a state that has features of “suspended animation”. Diapause helps the species survive extreme stress such as drought, by timing the birth of offspring to more environmentally favorable conditions (such as the rainy season). Diapause phenomena are widespread throughout the animal kingdom, including mammals (for example, in roe deer and bats, which helps the species survive winter [315, 316]). *C. elegans* also has several diapause-like states—notably the alternative developmental state called “dauer”—which help the species survive a dearth of food [317]. It is interesting to note that in *C. elegans*, the regulatory network underlying the dauer state shares many components with that underlying aging [318, 319]. In *C. elegans* or *Drosophila*, the period of time spent in diapause does not impact lifespan when these individuals reach adulthood (compared to individuals that did not go into diapause) [320–322]. This suggests that either no aging takes place during diapause or the damage caused by aging during this phase is corrected at the exit from diapause [323]. Thus, studying diapause could not only offer insight into the genetic network that regulates lifespan but also provide new ideas for prevention of damage accumulation or erasure of damage. However, due to the low embryo accessibility in mammals, diapause is vastly understudied in vertebrates. With its short lifespan and high embryo number and accessibility, the turquoise killifish is uniquely well-suited to study the relationship between diapause and aging and to understand how features of “suspended animation” could be harnessed.
